# Designing Programmable Peptide Nucleic Acid‐based Nanovaccines for Anticancer Immune Activation

**DOI:** 10.1002/smll.202505605

**Published:** 2025-11-07

**Authors:** Yanyu Huang, Cuiqing Huang, Sakshi Pandita, Chon Man Ieong, Yongheng Wang, David Wang, Jifeng Chen, Victorio Jauregui‐Matos, Alessandra Maria Arabelle Beelen, Ya‐Ping Shiau, Shiqi Tang, Junwei Zhao, Qiufang Zong, Menghuan Tang, Zhaoqing Cong, Yuanpei Li, Peter A. Beal, Sheila S. David, Aijun Wang, Duo Wang, Zeyu Xiao, Kit S. Lam

**Affiliations:** ^1^ Department of Biochemistry and Molecular Medicine University of California Davis, Sacramento CA 95817 USA; ^2^ The Guangzhou Key Laboratory of Molecular and Functional Imaging for Clinical Translation The First Affiliated Hospital of Jinan University Guangzhou 510632 China; ^3^ Center of Interventional Radiology & Vascular Surgery Department of Radiology Zhongda Hospital Medical School Southeast University Nanjing 210009 China; ^4^ Guangdong Women and Children Hospital Guangzhou 511442 China; ^5^ Radiology Department Centro Hospitalar Conde de São Januário Estrada do Visconde de S. Januário Macau Macao SAR 999078 China; ^6^ Department of Biomedical Engineering University of California Davis, Sacramento CA 95817 USA; ^7^ Department of Chemistry University of California Davis CA 95616 USA

**Keywords:** cancer immunotherapy, dendritic cells, melanoma, nanovaccine, peptide nucleic acid

## Abstract

Targeted delivery of antigens and adjuvants to the immune cells without eliciting uncontrolled inflammation is a major challenge in cancer vaccine development. Here, a highly versatile and programmable peptide nucleic acid (PNA)‐based vaccine nanoplatform (PVN) is reported to elicit a robust anti‐tumor immune response against B16‐OVA syngeneic melanoma model. The PVN is built on an 11‐mer PNA scaffold, enabling efficient “one‐pot” loading of a PNA‐modified ovalbumin antigenic peptide (SIINFEKL), CpG adjuvant, and a PNA‐derivatized LLP2A ligand (an immune cell and melanoma cell targeting ligand). Super‐resolution fluorescence imaging reveals the spatial arrangement of OVA_8_ within LP_10‐12_[*OVA_8_/CpG/LLP2A*], while circular dichroism spectroscopy confirmsparalleled binding of complementary PNA strands in LP_11_[*OVA_8_/CpG/LLP2A*]. LLP2A displayed on PVNs target activated α4β1 integrin expressed by immune and melanoma cells, boosting antigen presentation by dendritic cells and eliciting strong CD8+T cell   and natural killer cell responses. This amplified antitumor immune response leads to significant tumor regression and prolonged survival of mice bearing syngeneic B16‐OVA melanoma. The modular nature and versatility of PVN allow convenient one‐pot assembling of peptide antigens, immunomodulators, immune cell and tumor cell targeting ligands, making it practical for the custom design and preparation of personalized cancer vaccines.

## Graphical abstract

This study introduces a first‐of‐its‐kind peptide nucleic acid‐based vaccine nanoplatform (PVN) for precision cancer immunotherapy. PVN enables programmable, enzyme‐resistant, and targeted delivery of antigens and adjuvants, enhancing antitumor immunity. With LLP2A for improved antigen presentation, it elicits potent CD8+T and natural killer cell responses, driving robust melanoma regression and pioneering a new avenue for personalized cancer vaccines.

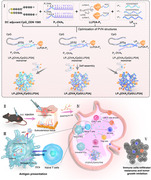

## 1 Introduction

Cancer immunotherapies, including checkpoint blockade antibodies, are now widely used in the clinic. While these therapies have proven highly effective against several cancer types and have become essential in modern treatment regimens, response rates can vary greatly among different cancer types.^[^ ^1^, ^2^ ^]^ Although only a few cancer vaccines have been approved by the U.S. Food and Drug Administration, many others are in preclinical and clinical development. These vaccines employ various modalities, such as cell‐based vaccines (using whole cells or cell fragments), peptide‐based vaccines (both chemically synthesized and biosynthetic), viral‐based vaccines, and nucleic acid‐based vaccines (e.g., DNA and mRNA vaccines).^[^ ^3^, ^4^, ^5^ ^]^ Vaccines can be administered as monotherapies or in combination with conventional treatments like chemotherapy, radiotherapy, and surgery.^[^ ^6^ ^–^ ^8^ ^]^

One of the major challenges in cancer vaccine development is the reliable delivery of antigens and adjuvants to the appropriate locations in the body, where immune responses can be effectively elicited, while also controlling unwanted inflammation.^[^ ^9^, ^10^, ^11^ ^]^ Strategies to overcome these challenges include the design of robust delivery platforms that protect the antigens and adjuvants from in vivo enzymatic degradation before they reach their target sites. Additionally, the inclusion of targeting ligands can help engage professional antigen‐presenting cells (APCs), thereby promoting more specific immune recognition. Although DNA origami and RNA‐based architectures have been used as scaffolds for such nanoplatforms^[^ ^12^, ^13^, ^14^ ^]^, few studies have exploited the use of peptide nucleic acids (PNAs) in constructing these nanostructures.

PNA is a synthetic analog of DNA in which the ribose phosphate backbone has been replaced by a polyamide chain.^[^ ^15^ ^]^ PNA's unique structure exhibits several advantages over conventional oligonucleotides: 1) PNA binds to complementary DNA, RNA, or PNA with higher affinity and greater specificity than DNA or RNA because of its uncharged polyamide backbone;^[^ ^16^ ^]^ 2) PNA is more stable than DNA or RNA as they are resistant to nucleases and proteases.^[^ ^17^ ^]^ Thus, the lifetime of PNAs is extended both in vivo and *in vitro*. 3) PNAs are not recognized by polymerases and therefore cannot be directly used as primers or copied, which minimizes the risks of random genome replication in vivo. 4) PNA can be hybridized with the complementary DNA/RNA/PNA independence of ionic strength, which leads to higher stringency and higher affinity to complementary nucleotides.^[^ ^18^ ^]^ The unique physicochemical properties of PNA molecules and their compatibility with peptide and chemical synthesis allow one to custom design a scaffold capable of integrating various biological and chemical components, such as oligonucleotides, peptides, immunostimulants, small molecules, and dyes, together to form a cancer vaccine, affording high versatility and biocompatibility.

Here, we report on the development of a robust PNA‐based vaccine nanoplatform (PVN) that not only can assemble antigen/adjuvant payloads and protect them from degradation by extracellular ribonucleases and proteases but also facilitates their efficient transport to the tumor‐draining lymph nodes (TDLNs). The vaccine consists of several components, some of which have a short PNA sequence to promote hybridization of the therapeutic payloads with the PNA scaffold: 1) The PNA‐SIINFKL peptide conjugate. SIINFEKL (i.e., OVA_8_) is a chicken ovalbumin epitope that serves as the T cell‐dependent antigen. 2) The well‐known immunomodulator ODN 1585 (a Class A CpG oligonucleotide and toll‐like receptor 9 agonist) can improve the immune response of antigen‐presenting cells.^[^ ^19^ ^]^ 3) The PNA‐LLP2A conjugate. LLP2A, previously identified by our laboratory via one‐bead, one‐compound combinatorial technology^[^ ^20^ ^]^, is a high‐affinity, high‐specificity peptidomimetic compound that binds activated α4β1 integrin expressed on bone marrow dendritic cells (BMDC), T cells, and also B16‐F10 melanoma cells. It promotes in vivo engagement between DCs and T cells as well as melanoma cells.

**Scheme 1:**
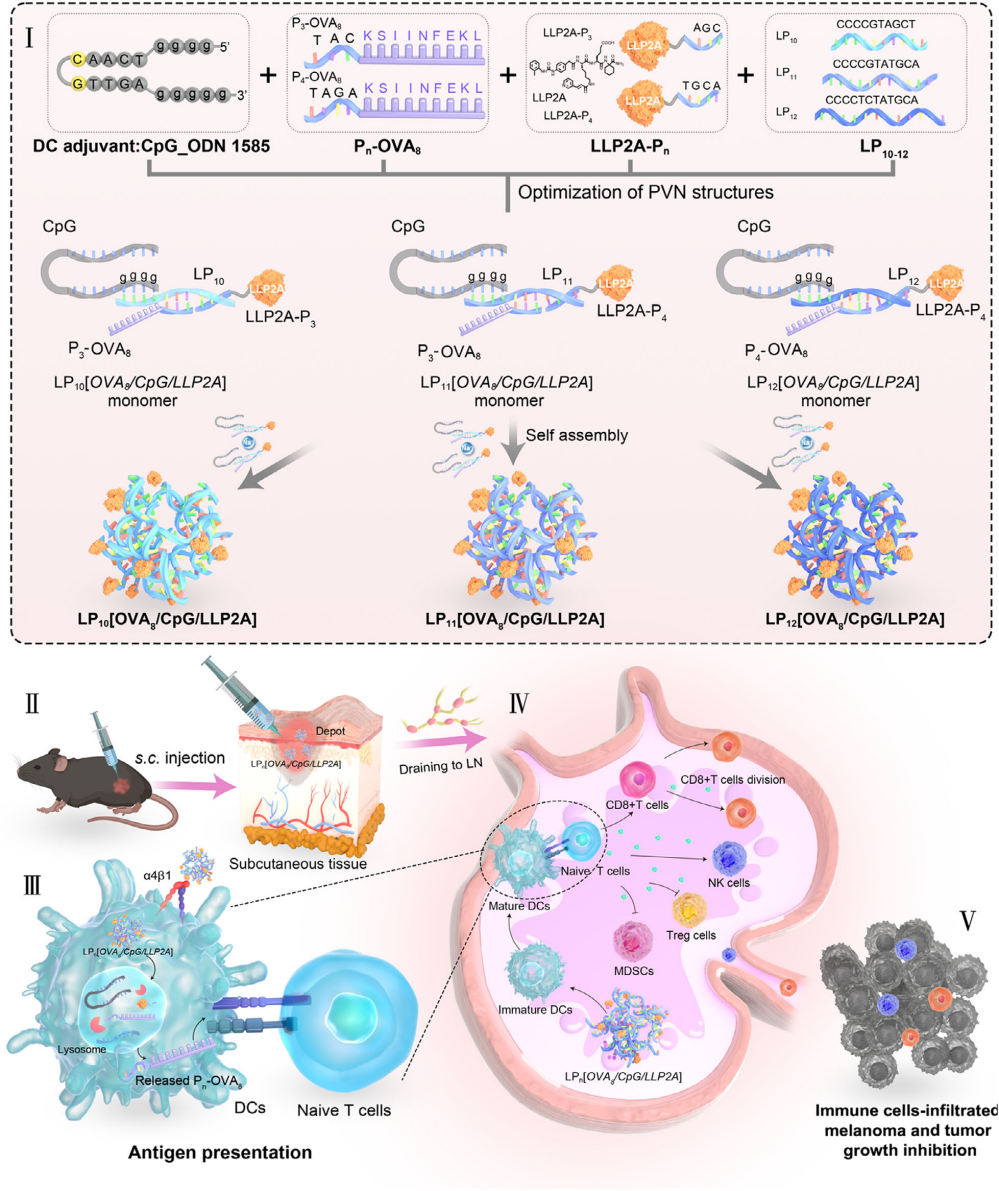
Schematic representation of the design, characterization, and utilization of the antigen/adjuvant‐assembled PNA‐based nanovaccine for anti‐melanoma therapy. I) Various lengths of LPs are hybridized with the complementary P_n_‐OVA_8_, CpG, and LLP2A‐P_n_ to form a PNA‐based nanovaccine. II) The PNA‐based nanovaccine, i.e., LP_n_[*OVA_8_/CpG/LLP2A*], is administered subcutaneously to mice bearing B16‐OVA melanoma. It effectively transports antigens and adjuvants to draining LNs (DLNs), III) where they accumulate within APC lysosomes. LLP2A displayed by LP_n_[*OVA_8_/CpG/LLP2A*] promotes the engagement between DC and T cells, thus facilitating DC maturation and antigen presentation to T cells. IV) This process triggers a potent, antigen‐specific CD8+ cytotoxic T lymphocyte response and NK activation, while simultaneously suppressing the function of myeloid‐derived suppressor cells (MDSCs) and regulatory T cells (Tregs). V) The potent anti‐immune response elicited by the optimized nanoscaffold, i.e., LP_11_[*OVA_8_/CpG/LLP2A*], significantly inhibited melanoma growth.

To facilitate the self‐assembly of the cancer vaccine, a short PNA sequence (i.e., P_n_, wherein *n* = number of the PNA monomers) was added to the N‐terminus of the OVA_8_ peptide to form P_n_‐OVA_8_, and to the carboxyl end of LLP2A to form LLP2A‐P_n_ (**Scheme** **1**). The sequences of these short PNAs were chosen such that they can be, together with CpG (aka ODN 1585), hybridized specifically in a “one‐pot” manner to the 10–12mer PNA scaffold via Watson‐Crick pairing to form PVN nanoconstructs displaying OVA_8_, CpG, and LLP2A (referred to as LP_n_[*OVA_8_/CpG/LLP2A*]). Of the three nanoconstructs (*n* = 10, 11, and 12) evaluated, LP_11_[*OVA_8_/CpG/LLP2A*] was found to be the most effective. It efficiently amplified a robust cytotoxic CD8+T cell and natural killer (NK) cell immunity in vivo, resulting in significant tumor regression and prolonged survival of the syngeneic B16‐OVA melanoma model in fully immunocompetent mice. This PVN platform, because of its simplicity and modularity, can be easily adapted to the development of personalized cancer vaccines.

## 2 Results and Discussion

### 2.1 Chemical Characterization of LP_n_[*OVA_8_/CpG/LLP2A*]

In this study, the antigen epitope OVA_8_, adjuvant CpG, and targeting ligand LLP2A were hybridized with the complementary linkage PNA scaffold (LP_n_) through the Watson‐Crick base pairing principle. To achieve that, we introduced a short PNA sequence (3‐mer or 4‐mer) to the N‐terminus of OVA_8_ and the C‐terminus of LLP2A via solid‐phase synthesis. For clarity, we will use the following nomenclature for the self‐assembled PNA‐based nanostructures. For example, LP_11_[*OVA_8_/CpG/LLP2A*] denotes a linkage PNA scaffold of 11‐mer (LP_11_), with the payload components italicized and placed inside the brackets. In this case, the three payloads are LLP2A‐PNA_4_ conjugate (abbreviated as *LLP2A*), PNA_3_‐OVA_8_ conjugate (abbreviated as *OVA_8_*), and CpG. OVA_8_, CpG, and LLP2A were mixed with LP_n_, and the ratio of OVA_8_, CpG, and LLP2A to LP_n_ was determined by HPLC and spectrophotometer (Table S1, Supporting Information).

**Figure 1:**
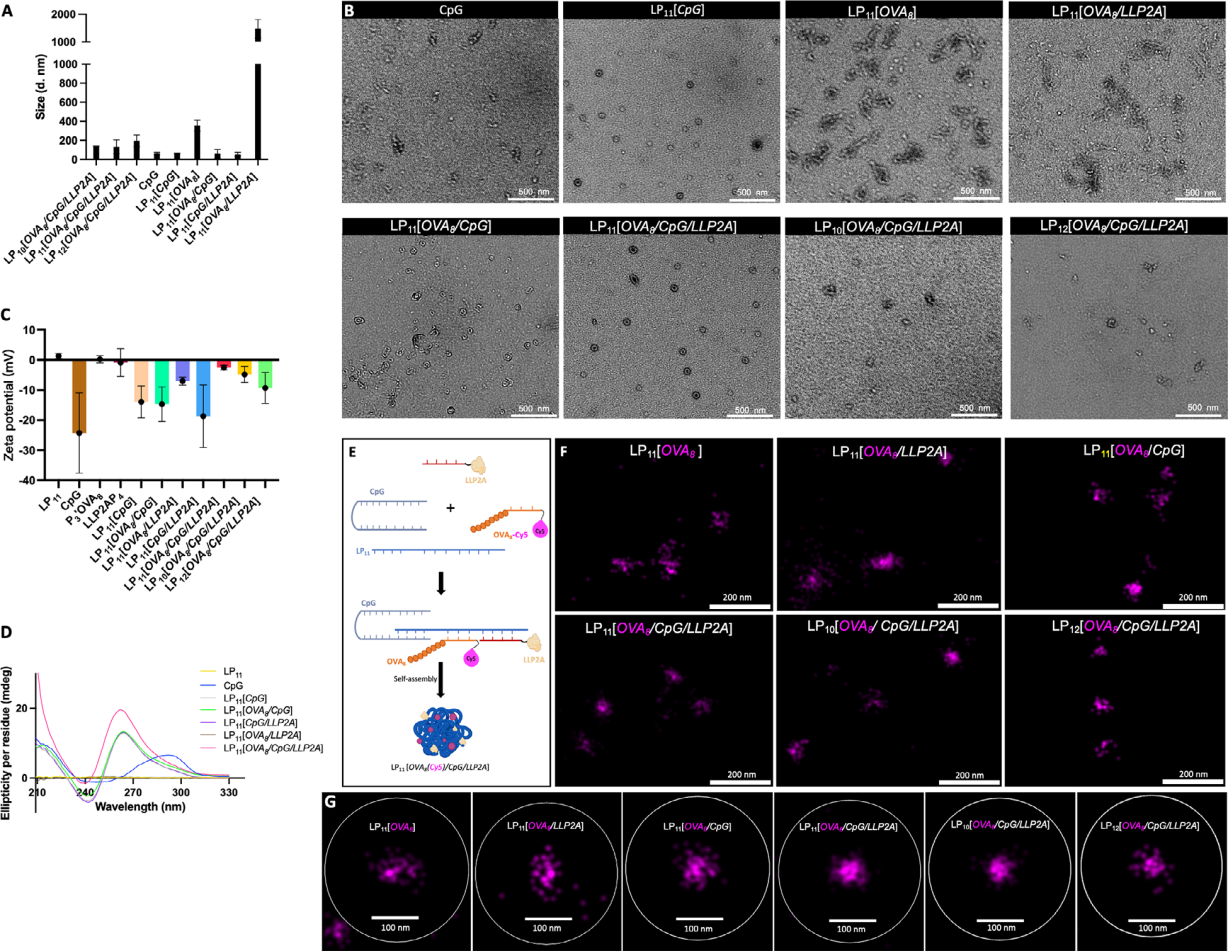
Physicochemical characterization of LP_n_[*OVA_8_/CpG/LLP2A*]. A) Size distribution of different complete PVNs (LP_n_[*OVA_8_/CpG/LLP2A*]) and selected incomplete PVNs in H_2_O. Data presented as mean ± SD, *n* = 3. B) TEM images of CpG, LP_11_[*CpG*], LP_11_[*OVA_8_*], LP_11_[*OVA_8_/CpG*], LP_11_[*OVA_8_/LLP2A*], LP_11_[*OVA_8_/CpG/LLP2A*], LP_10_[*OVA_8_/CpG/LLP2A*] and LP_12_[*OVA_8_/CpG/LLP2A*], scale bar = 500 nm. C) Zeta potential of LP_n_[*OVA_8_/CpG/LLP2A*] in H_2_O. Data presented as mean ± SD, *n* = 3. D) CD spectra of LP_11_, CpG, LP_11_[*CpG*], LP_11_[*OVA_8_/CpG*], LP_11_[*CpG/LLP2A*], LP_11_[*OVA_8_/LLP2A*], LP_11_[*OVA_8_/CpG/LLP2A*], containing equivalent concentrations of CpG, OVA_8_, and/or LP_11_ at 30 µm. E) Schematic illustration of the assembly of LP_11_[*OVA_8_(Cy5)/CpG/LLP2A*]. OVA_8_ was conjugated with Cy5 at the N’ terminus through click chemistry. F) Super resolution images of LP_11_[*OVA_8_*], LP_11_[*OVA_8_/CpG*], LP_11_[*OVA_8_/LLP2A*], LP_11_[*OVA_8_/CpG/LLP2A*], LP_10_[*OVA_8_/CpG/LLP2A*] and LP_12_[*OVA_8_/CpG/LLP2A*]. Scale bar = 200 nm. G) Representative images of an individual nanoparticle in different groups. Scale bar = 100 nm.

The size and the dispersity of each of the three nanoconstructs in water were determined by Malvern zetasizer. As shown in **Figure** **1**A, the average hydrodynamic size of LP_11_[*OVA_8_/CpG/LLP2A*] (135.3 nm ± 41.01) was similar to that of LP_10_[*OVA_8_/CpG/LLP2A*] (135.5 nm ± 5.5) and smaller than that of LP_12_[*OVA_8_/CpG/LLP2A*] (195.1 nm ± 61.1). The morphology of LP_10,11,12_[*OVA_8_/CpG/LLP2A*] exhibited a globular structure as shown in the transmission electron microscope (TEM) images of Figure 1B. To better understand how PVN nanoparticles are formed, we used LP_11_[*OVA_8_/CpG/LLP2A*] as an example and analyzed the hydrodynamic size, morphology, and spatial distribution of its components in various LP_11_‐based incomplete PVNs. It is well‐known that Class A CpG ODNs can self‐assemble into nanosized multimers because of their palindromic and poly‐guanine (G) tails.^[^ ^19^, ^21^, ^22^ ^]^ As shown in Figure 1A, the hydrodynamic size of CpG was 62.3 ± 14.2 nm, with a fibrinous morphology in the nanocomplex (Figure 1B). To determine whether G‐quadruplex structures formed in the CpG ODN nanoclusters, we used nuclear magnetic resonance (NMR) to detect the CpG structure. The NMR spectrum of CpG (Figure S1, Supporting Information) showed no characteristic imino peaks of G‐quadruplex between 10.0 and 12.5 ppm, indicating that the nanosized CpG was not assembled via G‐quadruplex formation. Mixing G‐rich CpG with C‐rich LP_11_ to form LP_11_[*CpG*] resulted in a globular structure with an average size of 63.5 ± 5.4 nm. Furthermore, hybridization with CpG, P_3_‐OVA_8_, and LLP2A‐P_4_ payloads increased the size of the final nanoparticle to 132.6 ± 71.7 nm.

We next determined the zeta potential of the prepared PVN. Typically, the negatively charged ribose backbone of DNA and the neutrally charged N‐(2‐aminoethyl) glycine backbone of PNA confer a negative charge to DNA and a neutral charge to PNA. As expected, the zeta potential of CpG DNA was negative (−24.3 ± 13.3 mV) while the zeta potential of PNA and PNA‐peptide hybrids (e.g., LP_11_, P_3_‐OVA_8_, LLP2A‐P_4_) was each found to approach 0 mV (Figure 1C; Figure S2, Supporting Information). The zeta potential values of LP_10, 11, 12_[*OVA_8_/CpG/LLP2A*] and the incomplete LP_11_ nanostructures were found to remain slightly negative.

PNA can bind to complementary oligonucleotides in both parallel and antiparallel orientations.^[^ ^23^ ^]^ To further understand the assembling process among the CpG DNA, the two PNA‐peptide sequences, and LP_11_ components in LP_11_[*OVA_8_/CpG/LLP2A*], we prepared various incomplete LP_11_ nanostructures and determined their circular dichroism (CD) spectra. As shown in Figure 1D, the CD spectra of LP_11_, LP_11_[*OVA_8_*], LP_11_[*LLP2A*], and LP_11_[*OVA_8_/LLP2A*] were nearly flat, indicating a lack of well‐defined secondary structures, which can be ascribed to the achiral structure of PNA.^[^ ^24^ ^]^ In contrast, the CD spectrum of CpG DNA showed a positive band at 295 nm and a negative band at 243 nm, suggesting that CpG adopted a B‐form helix.^[^ ^25^ ^]^ Interestingly, a maxima at ≈263 nm, a positive band at 210 nm, and a minimum at 243 nm were observed in the spectra of the three DNA‐PNA complexes, i.e., LP_11_[*CpG*], LP_11_[*OVA_8_/CpG*], and LP_11_[*OVA_8_/CpG/LLP2A*], suggesting that PNA bound to complementary oligonucleotides in a parallel orientation in these nanostructures.^[^ ^26^ ^]^

To visualize the spatial distribution of OVA_8_ within the nanostructures, super‐resolution fluorescent images of LP_n_[*OVA_8_/CpG/LLP2A*] were captured under direct stochastic optical reconstruction microscopy (dSTORM). Cy5 was conjugated at the N’ terminus of OVA_8_‐P_n_ (*n* = 3,4) by click chemistry to localize OVA_8_ within the PVN nanostructures (Figure 1E). The morphology of the resulting LP_11_[*OVA_8_*] and LP_11_[*OVA_8_/LLP2A*] exhibited a scattered arrangement, while the introduction of CpG to LP_11_[*OVA_8_*] and LP_11_[*OVA_8_/LLP2A*] has led to a decrease in the size of the multimer into more compact nanoparticles, which can be ascribed to the self‐assembly among poly G‐tails of CpG.^[^ ^22^ ^]^ A similar pattern can be seen in the structure of LP_10_[*OVA_8_/CpG/LLP2A*] and LP_12_[*OVA_8_/CpG/LLP2A*] (Figure 1F). These findings were consistent with the TEM images shown in Figure 1B. The representative individual nanocluster image in each group provided a magnified view of the spatial distribution of OVA_8_(Cy5), as illustrated in Figure 1G.

### 2.2 LP_n_[*OVA*_*8*_ */CpG/LLP2A*] Can Augment Anticancer Immunity In Vitro

**Figure 2:**
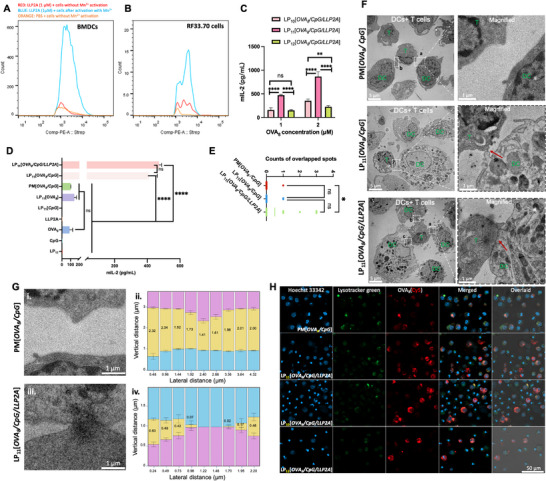
DC activation and antigen presentation mediated by LP_n_[*OVA_8_/CpG/LLP2A*] *in vitro*. Binding efficacy of LLP2A with BMDCs A) and RF33.70 cells B), respectively. α4β1 integrins on the surface of RF33.70 and BMDCs were pre‐activated with 1 mM Mn^2+^ before incubating with biotinylated LLP2A and then Strepavidin‐PE. C) LP_n_[*OVA_8_/CpG/LLP2A*]‐triggered IL‐2 expression from the co‐culture of RF33.70 cells and the activated BMDCs after 16 h. BMDC was pretreated with LP_n_[*OVA_8_/CpG/LLP2A*] containing 1 and 2 µm OVA_8,_ respectively, for 16 h. “ns” denotes not significant, ^**^ *p* < 0.01, ^***^ *p* < 0.001, ^****^ *p* < 0.0001, 2‐way ANOVA. Data presented as mean ± SD, *n* = 3. D) IL‐2 level of the co‐culture of RF33.70 cells and activated BMDCs after 16 h. BMDCs were pretreated with 1 µM LP_11_[*OVA_8_/CpG/LLP2A*], individual component or incomplete nanoformulations  for 16 h. “ns” denotes not significant, ^****^ *p* < 0.0001, one‐way ANOVA with Tukey's posthoc test. Data presented as mean ± SD, *n* = 3. E) Counts of DC‐T contact spots from ten TEM images after different treatments. BMDCs were pretreated with a physical mixture of OVA_8_ and CpG, LP_11_[*OVA_8_/CpG*], and LP_11_[*OVA_8_/CpG/LLP2A*] for 16 h, respectively, followed by co‐culturing with RF33.70 cells for 16 h. “ns” denotes not significant, *P<0.05, one‐way ANOVA with Tukey's posthoc test, *n* = 10. F) Representative TEM images of DC‐T‐cell contact mediated by physical mixture of OVA_8_ and CpG, LP_11_[*OVA_8_/CpG*] and LP_11_[*OVA_8_/CpG/LLP2A*] respectively, (scale bar = 5 µm). The magnified images depict the gaps between DC and T cells in white dotted boxes (scale bar = 1 µm). The close‐contact zones between DC and RF33.70 cells are denoted by the red arrows. G) i. Enlarged TEM image of *Zone a* from the TEM image of PM[*OVA_8_/CpG*] in Figure 2F, Scale bar = 1 µm; ii. Quantification of the distance between RF33.70 cells and BMDCs pretreated with PM[*OVA_8_/CpG*], *n* = 98 rows of pixels. iii. Enlarged TEM image of *Zone b* from the TEM image of LP_11_[*OVA_8_/CpG/LLP2A*] in Figure 2F, Scale bar = 1 µm; iv. Quantification of the distance between RF33.70 cells and BMDCs pretreated with LP_11_[*OVA_8_/CpG/LLP2A*], *n* = 98 rows of pixels. H) Intracellular trafficking of LP_n_[*OVA_8_* (Cy5)/*CpG/LLP2A*] (*n* = 10, 11, 12) or physical mixture of OVA_8_(Cy5) and CpG in BMDCs. Cy5 was conjugated at the N'‐terminus of OVA_8_ by click chemistry. The lysosomes were stained with lysotracker green, and the nucleus was stained with Hoechst 33 342. Scale bar = 50 µm.

α4β1 integrin is reported to be highly expressed on the surface of various immune cells, including T cells, B cells, DC, monocyte/macrophage, NK cells, and neutrophils.^[^ ^27^, ^28^ ^]^ It plays a key role in promoting immune cell migration (e.g., DCs, T cells, B cells, and macrophages), supporting the development of T and B cells, and enhancing the adhesion of NK cells and neutrophils.^[^ ^29^, ^30^ ^]^ We previously reported using the “one‐bead one‐compound” combinatorial library method to develop LLP2A, a high‐affinity and high‐specificity peptidomimetic ligand against activated α4β1 integrin displayed on the surface of activated normal lymphocytes and lymphoma cells.^[^ ^20^ ^]^ Using flow cytometry, we have determined that LLP2A could bind to BMDCs and RF33.70 cells (a T‐cell hybridoma cell line developed by Dr. Kenneth Rock's lab) following Mn^2+[^ ^31^ ^]^ treatment to activate α4β1 integrin (**Figure** **2**A,B). Furthermore, pre‐incubation with a saturating concentration of LLP2A to block the α4β1 integrin on BMDCs and RF33.70 cells markedly reduced subsequent LLP2A binding to these cells (Figure S3, Supporting Information), demonstrating that LLP2A binds to BMDCs and RF33.70 cells via specific targeting to α4β1 integrin. We expect that LLP2A displayed on the surface of LP_n_[*OVA_8_/CpG/LLP2A*] would promote physical engagement between BMDCs and RF33.70 T‐cell hybridoma, resulting in significant amplification of T‐cell activation.^[^ ^32^ ^]^ Incidentally, α4β1 integrin has also been found to be overexpressed in melanoma;^[^ ^33^, ^34^ ^]^ thus, LLP2A can serve as a targeting ligand against both immune cells and melanoma cells.

To understand the anticancer immunity mechanisms of the PVN platform, the activation of APC induced by LP_n_[*OVA_8_/CpG/LLP2A*] at different configurations and concentrations was evaluated *in vitro*. The BMDCs were pretreated with LP_10_[*OVA_8_/CpG/LLP2A*], LP_11_[*OVA_8_/CpG/LLP2A*], or LP_12_[*OVA_8_/CpG/LLP2A*], followed by co‐incubation with T‐cell hybridoma, i.e., RF33.70 cell line, for 24 h. The RF33.70 cell line is a CD8+ T cell hybridoma with specificity for the OVA peptide SIINFEKL presented on H2‐Kb,^[^ ^35^ ^]^ eliciting IL‐2 production upon stimulation.^[^ ^36^, ^37^ ^]^ As shown in Figure 2C, a significantly higher IL‐2 level was detected in the LP_11_[*OVA_8_/CpG/LLP2A*]‐treated cells compared to that of the LP_10_[*OVA_8_/CpG/LLP2A*]‐ and LP_12_[*OVA_8_/CpG/LLP2A*]‐treated cells, in a dose‐dependent manner. This result demonstrated that LP_11_[*OVA_8_/CpG/LLP2A*] outperformed its counterparts, e.g., LP_10_[*OVA_8_/CpG/LLP2A*] and LP_12_[*OVA_8_/CpG/LLP2A*], for stimulating a higher immune response toward DC and T cells *in vitro *(Figure 2C). Therefore, LP_11_[*OVA_8_/CpG/LLP2A*] was chosen as the PVN platform to explore the immune activation contributed by each component. Here, we use PM[*OVA_8_/CpG*] to denote a physical mixture of OVA_8_ and CpG. We found that IL‐2 production by cells was barely induced upon treatment with free LP_11_, CpG, or LLP2A. In contrast, free OVA_8_ (i.e._,_ SIINFEKL peptide), PM[*OVA*_*8*_ */* *CpG*] and LP_11_[*OVA*_*8*_] induced only low level of IL‐2, whereas LP_11_[*OVA_8_/CpG*] elicited 4.1 times higher IL‐2 levels than PM[*OVA_8_/CpG*]. Notably, LP_11_ co‐loaded with OVA_8_, CpG, and LLP2A significantly induced IL‐2 production compared to other groups. (Figure 2D). We believe the high potency of LP_11_[*OVA_8_/CpG/LLP2A*] in inducing IL‐2 production can be ascribed to the targeting effect of LLP2A toward α4β1 integrin expressed by BMDCs. To validate this mechanism, we detected the IL‐2 level in the co‐culturing of BMDCs and RF33.70 cells after blocking BMDCs with an excess amount of LLP2A‐P_4_ before exposure to LP_11_[*OVA_8_/CPG/LLP2A*]. Our results showed that IL‐2 expression was reduced in the LLP2A‐P_4_–blocked BMDC group compared with the group without LLP2A‐P4 blockade, which further demonstrated that the interaction between LLP2A and α4β1 integrin facilitates DC–T cell engagement, as reflected by IL‐2 production. (Figure S4, Supporting Information). Consistent with these results above, LP_11_[*OVA_8_/CpG/LLP2A*] was also found to be taken up by DCs more efficiently than PM[*OVA_8_/CpG*] and LP_11_[*OVA_8_/CpG*] by 2 and 8 h of incubation (Figure S5, Supporting Information). On the other hand, LP_12_[*OVA_8_/CpG/LLP2A*] exerted significantly lower uptake efficiency than LP_11_[*OVA_8_/CpG/LLP2A*] and LP_10_[*OVA_8_/CpG/LLP2A*, which could be attributed to their bigger size.

Encouraged by the enhanced *in vitro* immune response elicited by LP_11_[*OVA_8_/CpG/LLP2A*], we proceeded to visualize the DC‐T cell interaction dynamics by examining the interface between LP_11_[*OVA_8_/CpG/LLP2A*]‐treated BMDCs and RF33.70 T cells with transmission electron microscopy (TEM). The DC‐T cell synapse is characterized by multiple finger‐like structures protruding from T cells into the cell body of the DC. At the focal protrusions, the plasma membranes of both cells come into direct contact and might correspond to T cell microvilli,^[^ ^38^ ^]^ which were presumed to facilitate TCR‐pMHC binding.^[^ ^39^ ^]^ Overlapped spots between BMDCs and RF33.70 cells were found to be the highest in LP_11_[*OVA_8_/CpG/LLP2A*] treated cells, compared to those cells treated with LP_11_[*OVA_8_/CpG*] or PM[*OVA_8_/CpG*]. For this experiment, ten TEM images for each treatment group were used in the analysis (Figure 2E). The representative TEM images (Figure 2F; Figure S6) did not reveal any T cell protrusions into the BMDC cell body in the PM[*OVA_8_/CpG*] treated cells, as reflected by the gaps between the BMDCs and T cells under high magnification. In contrast, close DC‐T cell contact was seen at the DC‐T cell interface after treatment with LP_11_[*OVA_8_/CpG*]. Moreover, more T cell protrusions were found firmly contacting BMDCs, as shown in dashed *Zones a‐c*. To further characterize the DC‐T cell contact sites, representative *Zone a* in the PM[*OVA_8_/CpG*] treatment group and *Zone b* in the LP_11_[*OVA_8_/CpG/LLP2A*] treatment group were selected and quantified. In the LP_11_[*OVA_8_/CpG/LLP2A*] treatment group, the vertical distance was zero in the middle of the lateral distance, while the intermembrane spacing of the middle was measured at 1.58 µm on average in the PM[*OVA_8_/CpG*] treatment group (Figure 2G). The close connection between DCs and T cells indicated more effective antigen presentation between LP_11_[*OVA_8_/CpG/LLP2A*]‐pretreated BMDCs and T cells, which could be ascribed to the enhanced binding affinity of LLP2A with the activated BMDCs.

Next, we measured the release rate of the antigen, OVA_8_, from LP_n_[(*OVA_8_/CpG/LLP2A*] at various pH levels to simulate different cellular compartments, including the cytosol (pH 7.4), lysosomes of immature DCs (pH 5.5), and lysosomes of matured DCs (pH 4.5). To quantify that, OVA_8_(Cy5) was substituted for OVA_8_ during the preparation of PVNs. As shown in Figure S8 (Supporting Information), the release rate of OVA_8_(Cy5) from all three PVN formulations, i.e., LP_10,11,12_[*OVA_8_/CpG/LLP2A*], followed a similar pattern, where OVA_8_(Cy5) was released faster from PVNs at acidic pH (pH 5.5 and pH 4.5) within the 72 h of incubation than at neutral pH 7.4. This is likely driven by pH‐dependent destabilization primarily from the disruption of guanine multimers through altered protonation and hydrogen bonding,^[^ ^40^ ^]^ which led to the dissociation between P_n_‐OVA_8_(Cy5) and LP_n_ bound by only 3–4 base pairs.

Furthermore, the stability of LP_10,11,12_[*OVA_8_/CpG/LLP2A*] under physiological and lysosomal conditions (pH 7.4, 5.5, and 4.5) was investigated by monitoring the particle size changes. LP_11_[*OVA_8_/CpG/LLP2A*] exhibited the greatest stability at pH 7.4, maintaining a size of 108.6–159.8 nm over four days before increasing to 245.7 nm by day six (Figure S9, Supporting Information), which is favorable for lymph node transport after *s.c*. administration. In contrast, LP_10_[*OVA_8_/CpG/LLP2A*] and LP_12_[*OVA_8_/CpG/LLP2A*] showed substantial instability at pH 7.4, with LP_10_[*OVA_8_/CpG/LLP2A*] size increasing to >800 nm within four days, and LP_12_[*OVA_8_/CpG/LLP2A*] exceeding 500 nm in a day, the latter attributed to increased PNA monomer content and hydrophobicity in physiological salt conditions. At acidic pH (5.5 and 4.5), LP_10,11_[*OVA_8_/CpG/LLP2A*] were slightly larger on day one but remained stable thereafter, and LP_12_[*OVA_8_/CpG/LLP2A*] consistently exceeded 700 nm. The larger size of LP_10,11,12_[*OVA_8_/CpG/LLP2A*] at these acidic pH levels is likely due to the instability of CpG multimers as mentioned above.

Next, we sought to monitor the intracellular trafficking of LP_n_[*OVA_8_/CpG/LLP2A*] after internalization by BMDCs via real‐time imaging of LP_11_[*OVA_8_/CpG/LLP2A*] containing Cy5‐labeled P_n_‐OVA_8_. The nucleus of BMDCs was stained with Hoechst 33342, and the lysosomes were stained with lysotracker green. As shown in Figure 2H, the Cy5 fluorescence signal of the LP_10‐12_[*OVA_8_/CpG/LLP2A*] treated cells was significantly stronger than that of PM[*OVA_8_/CpG*]‐treated cells, demonstrating that LP_n_[*OVA_8_/CpG/LLP2A*] was able to facilitate the cellular uptake of OVA_8_ in BMDCs. Importantly, the fluorescence signal of LP_10‐12_[*OVA_8_/CpG/LLP2A*] was found to co‐localize with lysotracker green after 8 h of incubation, indicating that LP_n_[*OVA_8_/CpG/LLP2A*] accumulated in the lysosome after internalization. The acidic microenvironment of the lysosome would facilitate the unwinding of the PNA nanovaccine and promote the release of payloads for immune activation.

### 2.3 LP_11_[*OVA_8_/CpG/LLP2A*]‐Augmented Antitumor Immunity for Growth Inhibition of Melanoma in a Syngeneic Model

**Figure 3:**
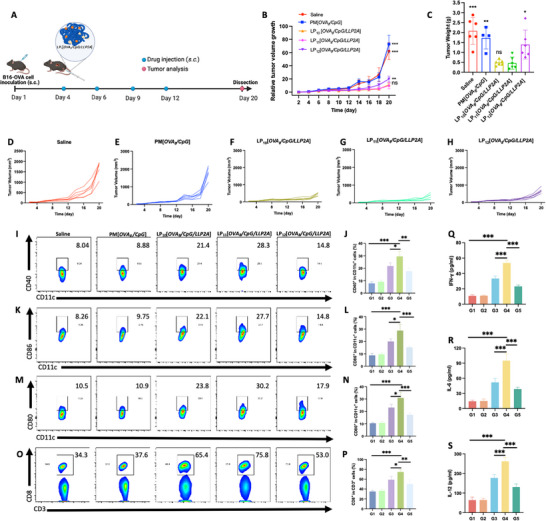
Antitumor efficiency of LP_n_[*OVA_8_/CpG/LLP2A*] in B16‐OVA_8_‐bearing mice. A) Schematic illustration of LP_n_[*OVA_8_/CpG/LLP2A*] inhibiting tumor growth in a B6‐OVA‐bearing tumor model. The mice were *s.c*.injected with LP_n_[*OVA_8_/CpG/LLP2A*], each containing an equivalent OVA_8_ concentration of 5 µM, on Days 4, 6, 9, and 12 following tumor inoculation. Tumors, blood, and major organs were collected when mice were sacrificed on Day 20. B) Tumor volume of B16‐OVA‐Luc bearing mice after various treatments for 20 days. Data are expressed as mean ± SD (*n* = 6). “ns” denotes “not significant”, ^**^ *p* < 0.01, ^***^ *p* < 0.001 versus LP_11_[*OVA_8_/CpG/LLP2A*] group, one‐way ANOVA with Tukey's posthoc test. C) Tumor weight of B16‐OVA‐Luc bearing mice on Day 20 for different treatment groups. Data are expressed as mean ± SD (*n* = 6). “ns” denotes “not significant”, ^*^ *p* < 0.05, ^**^ *p* < 0.01, ^***^ *p* < 0.001 versus LP_11_[*OVA_8_/CpG/LLP2A*] group, one‐way ANOVA with Tukey's posthoc test. Tumor volume curves of B16‐OVA‐bearing mice after treatment of D) saline, E) Physical mixture of *OVA_8_* and *CpG*, F) LP_10_[*OVA_8_/CpG/LLP2A*], G) LP_11_[*OVA_8_/CpG/LLP2A*], H) LP_12_[*OVA_8_/CpG/LLP2A*] for 20 days (*n* = 6). I) Representative flow cytometry scatter plots and histograms of the proportion of CD11c+CD40+BMDCs (I, J), CD11c+CD86+BMDCs K, L), CD11c+CD80+BMDCs M, N), CD3+CD8+T cells O, P) in DLN. Expression of IFN‐γ Q), IL‐6 R), and IL‐12 S) in plasma on Day 20 for different treatment groups (*n* = 3). G1: saline, G2: physical mixture of OVA_8_ and CpG, G3: LP_10_[*OVA_8_/CpG/LLP2A*], G4: LP_11_[*OVA_8_/CpG/LLP2A*], G5: LP_12_[*OVA_8_/CpG/LLP2A*]. ^*^ *p* < 0.05, ^**^ *p* < 0.01, ^***^ *p* < 0.001, one‐way ANOVA with Tukey's posthoc test.

Next, we studied the antitumor activity of LP_n_[*OVA_8_/CpG/LLP2A*] in a syngeneic B16‐F10‐OVA melanoma model, in which the mice were injected subcutaneously with tumor cells on day 1, followed by the nanovaccine given subcutaneously between the tumor and the draining lymph nodes on days 4, 6, 9, and 12 (**Figure** **3**A). As shown in Figure 3B–H, tumor size in both the untreated group and PM[*OVA_8_/CpG*]‐treated group exhibited a rapid increase by day 20. In contrast, tumor growth and weight were found to be greatly suppressed in the LP_10‐12_[*OVA_8_/CpG/LLP2A*] treated groups. The anti‐tumor immune potencies among the three PVN‐treated groups were found to be in the following descending order: LP_11_[*OVA_8_/CpG/LLP2A*] > LP_10_[*OVA_8_/CpG/LLP2A*] > LP_12_[*OVA_8_/CpG/LLP2A*]. We further investigated the immune cell profile of tumor tissues excised from treated mice. As illustrated in Figure 3I–P, all three PVNs elicited more robust anticancer immunity compared to PM[*OVA_8_/CpG*]. This enhanced immune response was evidenced by higher levels of DC maturation and CD8+ T cell expansion, as reflected by increased proportions of CD11c+CD40+, CD11c+CD86+, CD11c+CD80+ DCs, and CD3+CD8+ T cells. Notably, LP_11_[*OVA_8_/CpG/LLP2A*] induced significantly higher levels of DC maturation and CD8+ T cell proliferation than both LP_10_[*OVA_8_/CpG/LLP2A*] and LP_12_[*OVA_8_/CpG/LLP2A*] did. Moreover, LP_11_[*OVA_8_/CpG/LLP2A*] markedly elevated the levels of IFN‐γ, a cytokine typically released during CpG‐augmented CD8+ T cell activity. Additionally, it enhanced the secretion of immune cytokines by DCs, including both IL‐6 and IL‐12 (Figure 3Q–S).

Systemic toxicity of LP_n_[*OVA_8_/CpG/LLP2A*] was evaluated in both normal mice and mice bearing melanoma. As shown in Figure S10 (Supporting Information), the body weight of B16‐OVA‐bearing mice remained stable throughout the 20‐day treatment period with LP_n_[*OVA_8_/CpG/LLP2A*], indicating no significant adverse effects on the overall health of the animals. Furthermore, we assessed key biochemical profiles in the plasma and blood counts of healthy mice treated with LP_n_[*OVA_8_/CpG/LLP2A*] at Day 30. These markers, including white blood cell count (WBC), red blood cell count (RBC), platelet count (PLT), alanine aminotransferase (ALT), aspartate aminotransferase (AST), albumin (ALB), blood urea nitrogen (BUN), creatinine (CR), and uric acid (UA), showed no significant changes compared to the saline control group (Figure S11, Supporting Information). These findings strongly suggest that LP_10‐12_[*OVA_8_/CpG/LLP2A*] did not cause any significant systemic toxicity in vivo. Together, these results demonstrated that OVA_8_ and CpG, when nanoformulated as LP_10‐12_[*OVA_8_/CpG/LLP2A*], especially LP_11_[*OVA_8_/CpG/LLP2A*], were able to elicit a very strong anticancer immune response while maintaining a favorable safety profile with no significant systemic toxicity in vivo.

### 2.4 Mechanism of LP_11_[*OVA_8_/CpG/LLP2A*]‐Induced Antitumor Immunity

**Figure 4:**
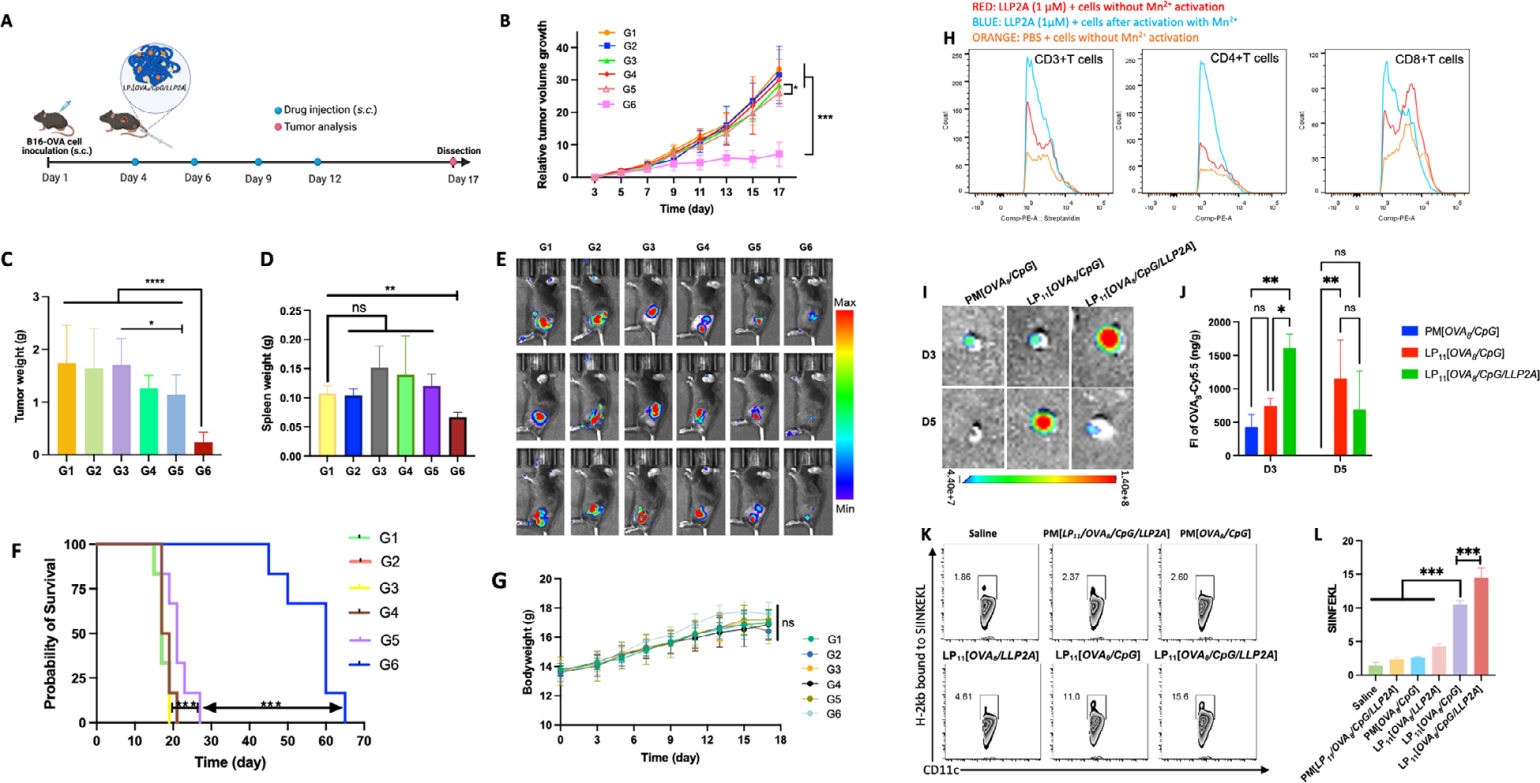
Antitumor efficiency of LP_11_‐based nanovaccine in B16‐OVA_8_‐bearing mice. A) Schematic illustration of LP_11_[*OVA_8_/CpG/LLP2A*] inhibiting tumor growth in a B6‐OVA‐bearing tumor model. The mice were *s.c*. injected with LP_11_[*OVA_8_/CpG/LLP2A*], individual component or incomplete nanoformulations, each containing 5 µm of OVA_8_ or CpG on Days 4, 6, 9, and 12 following tumor inoculation. Tumors, blood, and major organs were collected when mice were sacrificed on Day 17. B) Tumor volume of B16‐OVA‐Luc bearing mice after various treatments for 17 days (*n* = 7). ^*^ *p* < 0.05, ^***^ *P*<0.001, one‐way ANOVA with Tukey's posthoc test. Tumor weight C) and spleen weight D) of B16‐OVA‐Luc bearing mice on Day 17 for different treatment groups. “ns” denotes not significant, ^*^ *p* < 0.05, ^**^ *p* < 0.01, ^****^ *p* < 0.0001, one‐way ANOVA with Tukey's posthoc test. Data are expressed as mean ± SD (*n* = 7). E) Bioluminescence images of B16‐OVA‐Luc tumor‐bearing mice on Day 17 for different treatment groups (*n* = 3). F) Survival rate of B16‐OVA‐Luc bearing mice after various treatments for 17 days (*n* = 7). ^***^ *p* < 0.001, Logrank (Mantel‐Cox) test. G) Bodyweight of B16F10‐OVA‐bearing mice after various treatments for 17 days (*n* = 7). “ns” denotes not significant among different treatment groups. G1: Saline, G2: Physical mixture of LP_11_, P_3_‐OVA_8_, CpG and LLP2A‐P_4_, G3: LP_11_[*OVA_8_*], G4: LP_11_[*OVA_8_/LLP2A*], G5: LP_11_[*OVA_8_/CpG*], G6: LP_11_[*OVA_8_/CpG/LLP2A*]. H) Binding efficacy of LLP2A with CD3+T cells, CD4+T cells, and CD8+T cells. α4β1 integrins on the surface of CD3+T cells, CD4+T cells, and CD8+T cells were activated with 1 mM Mn^2+^ before incubating with 1 µM LLP2A (*n* = 3). I) Representative fluorescence images of TDLN from the B16‐OVA‐bearing mice after s.c. injection with physical mixture of OVA_8_(Cy5.5) and CpG, LP_11_[*OVA_8_* (Cy5.5)*/CpG*], or LP_11_[*OVA_8_* (Cy5.5)*/CpG/LLP2A*] on Days 3 and 5. J) Fluorescence of OVA_8_‐Cy5.5 in the TDLN from the B16‐OVA‐bearing mice after s.c. injection with physical mixture of OVA_8_(Cy5.5) and CpG, LP_11_[*OVA_8_* (Cy5.5)*/CpG*], or LP_11_[*OVA_8_* (Cy5.5)*/CpG/LLP2A*] for 3 days and 5 days (*n* = 3). “ns” denotes not significant, ^*^ *p* < 0.05, ^**^ *p* < 0.01, two‐way ANOVA with Tukey's posthoc test. Representative scatter plots K) and quantitative histogram L) of SIINFEKL expression on BMDCs in TDLN from the B16‐OVA‐bearing mice on Day 3 post s.c. injection with physical mixture of OVA_8_ and CPG, physical mixture of LP_11_, P_3_‐OVA_8_, CpG and LLP2A‐P_4_, LP_11_[*OVA_8_/LLP2A*], LP_11_[*OVA_8_/CpG*], or LP_11_[*OVA_8_/CpG/LLP2A*] (*n* = 3). Each treatment contains an equivalent OVA_8_ concentration of 5 µM. “ns” denotes not significant, ^***^ *P*<0.001, one‐way ANOVA with Tukey's posthoc test.

To elucidate the underlying mechanism of enhanced antitumoral effects of PVN platform, we compared the antitumor effects of LP_11_[*OVA_8_/CpG/LLP2A*], various LP_11_ based incomplete PVNs, and physical mixture of LP_11_, P_3_‐OVA_8_, LLP2A‐P_4_, and CpG but without going through the thermal dissociation and reannealing steps (PM[*LP_11_*/*OVA_8_/CpG/LLP2A*]), in a B16‐F10‐OVA‐Luc tumor model (**Figure** **4**A). As shown in Figure 4B and Figure S12 (Supporting Information), LP_11_[*OVA_8_/CpG*] exhibited a stronger anti‐tumoral effect compared to PM[*LP_11_*/*OVA_8_/CpG/LLP2A*], LP_11_[*OVA_8_*] and LP_11_[*OVA_8_/LLP2A*] over 17 days of treatment (4 injections). Notably, the incorporation of LLP2A into the nanovaccine formulation significantly enhanced its antitumor potency. LP_11_[*OVA_8_/CpG/LLP2A*] exhibited a 3.7 fold stronger tumor inhibitory effect compared to that of the LP_11_[*OVA_8_/CpG*]. Consistent with these findings, tumor weights in the LP_11_[*OVA_8_/CpG*] and LP_11_[*OVA_8_/LLP2A*] treated groups were smaller than those in the PM[*LP_11_*/*OVA_8_/CpG/LLP2A*] and LP_11_[*OVA_8_*] treated groups. More strikingly, the tumor weight in the LP_11_[*OVA_8_/CpG/LLP2A*] treated group was 4.7 fold lower than that of the LP_11_[*OVA_8_/CpG*] treated group (Figure 4C).

Spleen weight, which is associated with the cellular immune response and positively correlates with tumor weight in tumor‐bearing mice, was also assessed.^[^ ^41^, ^42^ ^]^ As depicted in Figure 4D, the spleen weight in the LP_11_[*OVA_8_/CpG/LLP2A*] treated melanoma‐bearing mice was significantly lower than that in other treatment groups, suggesting slower tumor progression in these mice.

Next, we used bioluminescence imaging to assess the tumor growth and therapeutic effect in living mice after 17 days of treatment. As shown in Figure 4E, while PM[*LP_11_*/*OVA_8_/CpG/LLP2A*] or LP_11_[*OVA_8_*] was able to elicit a modest decrease in tumor size, LP_11_[*OVA_8_/CpG/LLP2A*] was found to be much more efficacious. This anti‐tumor effect was evidenced by the markedly diminished luciferin bioluminescence signal observed in the tumor region of mice treated with LP_11_[*OVA_8_/CpG/LLP2A*].

The survival curves for all treatment groups, presented in Figure 4F, revealed striking differences in therapeutic efficacy. LP_11_[*OVA_8_/CpG*] showed a marginal improvement, extending the survival of B16‐F10‐bearing mice to 27 days compared to the PM[*LP_11_*/*OVA_8_/CpG/LLP2A*], LP_11_[*OVA_8_*], and LP_11_[*OVA_8_/LLP2A*] treated groups. However, LP_11_[*OVA_8_/CpG/LLP2A*] demonstrated a remarkable enhancement in therapeutic efficacy, significantly prolonging the survival of B16‐F10‐bearing mice to 65 days. This substantial increase in survival time underscores the superior antitumoral potency of the complete LP_11_[*OVA_8_/CpG/LLP2A*] nanovaccine formulation.

Next, we performed the terminal deoxynucleotidyl transferase dUTP nick‐end labeling (TUNEL) assay to detect cell death‐associated DNA fragmentation by endonucleases in tumor regions after treatments with the various PVNs. As shown in Figure S13 (Supporting Information), mice treated with LP_11_[*OVA_8_/CpG*] or LP_11_[*OVA_8_/LLP2A*] resulted in modest levels of apoptosis in the tumor tissues. In contrast, LP_11_[*OVA_8_/CpG/LLP2A*] was able to greatly enhance the level of apoptosis, as evidenced by stronger fluorescence in the tumor tissue. Moreover, the Ki67 (a proliferation marker) fluorescence was found to significantly decrease in the LP_11_[*OVA_8_/CpG/LLP2A*] treatment group compared to other treatment groups, further demonstrating the enhanced antitumor immunity capacity of LP_11_[*OVA_8_/CpG/LLP2A*] in melanoma regression. Of note, no significant weight loss was observed in mice across all treatment groups within the 17‐day period of therapy (Figure 4G), indicating the safety of the PVNs in vivo. These data collectively demonstrate that the combination of CpG, OVA_8_, and LLP2A in the LP_11_‐based nanoplatform results in enhanced antitumor efficacy compared to the individual components alone.

Subcutaneously (*s.c.)* or intramuscularly (*i.m.)* administered nanovaccine typically forms depots at the injection site before migrating to TDLNs, subsequently inducing a systemic antitumor effect.^[^ ^43^ ^]^ The efficiency of nanovaccines in infiltrating adjacent lymphatic vessels is significantly influenced by their physical and chemical properties, including softness, surface chemistry, dispersity, and size.^[^ ^43^ ^]^ Here, we hypothesized that LP_11_[*OVA_8_/CpG/LLP2A*] could efficiently accumulate in TDLNs because of their small size and the presence of LLP2A for specific targeting of immune cells. To investigate this hypothesis, the lymph node (LN)‐accumulation of LP_11_[*OVA_8_/CpG/LLP2A*] was monitored over time with *ex* *vivo* imaging after *s.c*. injection. Cy5.5 was conjugated to the N‐terminus of OVA_8_ through click chemistry, such that OVA_8_ can be conveniently monitored in vivo after administration. We have already demonstrated that LLP2A effectively binds to CD3+T cells, CD8+T cells, and CD4+T cells that had been pre‐activated with Mn^2+^ (Figure 4H). Thus, LP_11_[*OVA_8_/CpG/LLP2A*] displaying LLP2A is expected to facilitate its interaction with immune cells in TDLN, leading to triggering of antitumor immunity. The whole‐body imaging (Figure S14A, Supporting Information) showed that the OVA_8_(Cy5.5) fluorescence in PM[*OVA_8_/CpG*] treated mice was significantly weaker than that of LP_11_[*OVA_8_/CpG/LLP2A*] and LP_11_[*OVA_8_/CpG*] treated mice during the 5 days of observation, implying the fast elimination of free OVA_8_ peptide in vivo. In the ex vivo images of the LP_11_[*OVA_8_/CpG/LLP2A*] and LP_11_[*OVA_8_/CpG*] treated mice, a high OVA_8_‐Cy5.5 signal was seen in the lymph nodes and liver within 120 h of injection, implying that the PVNs (or possibly a small amount of dissociated OVA_8_‐Cy5.5) entered the lymphatic system and traveled to the TDLN after *s.c*. injection, and subsequently reached the liver via the systemic blood circulation.^[^ ^44^ ^]^ Notably, LP_11_[*OVA_8_/CpG/LLP2A*] exhibited faster accumulation in the TDLN compared to LP_11_[*OVA_8_/CpG*] (Figure 4I,J; Figure S14B–D, Supporting Information), as reflected by the stronger Cy5.5 fluorescence on day 3 after *s.c*. administration, whereas LP_11_[*OVA_8_/CpG*] reached its peak accumulation on day 5 post‐administration. This result could be explained by the targeting effect of LLP2A to immune cells, including CD8+T cells, CD4+T cells, and CD3+ T cells, which are enriched in the TDLN. On the other hand, the quantitative data showed a small amount of OVA_8_(Cy5.5) accumulated in the tumor in all groups, which could be attributed to systemic circulation and the lymphatic system. Overall, the expedited accumulation of LP_11_[*OVA_8_/CpG/LLP2A*] in lymph nodes is postulated to augment the efficiency of antigen processing by APCs, potentially facilitating the rapid induction of anti‐tumor immune responses.

**Figure 5:**
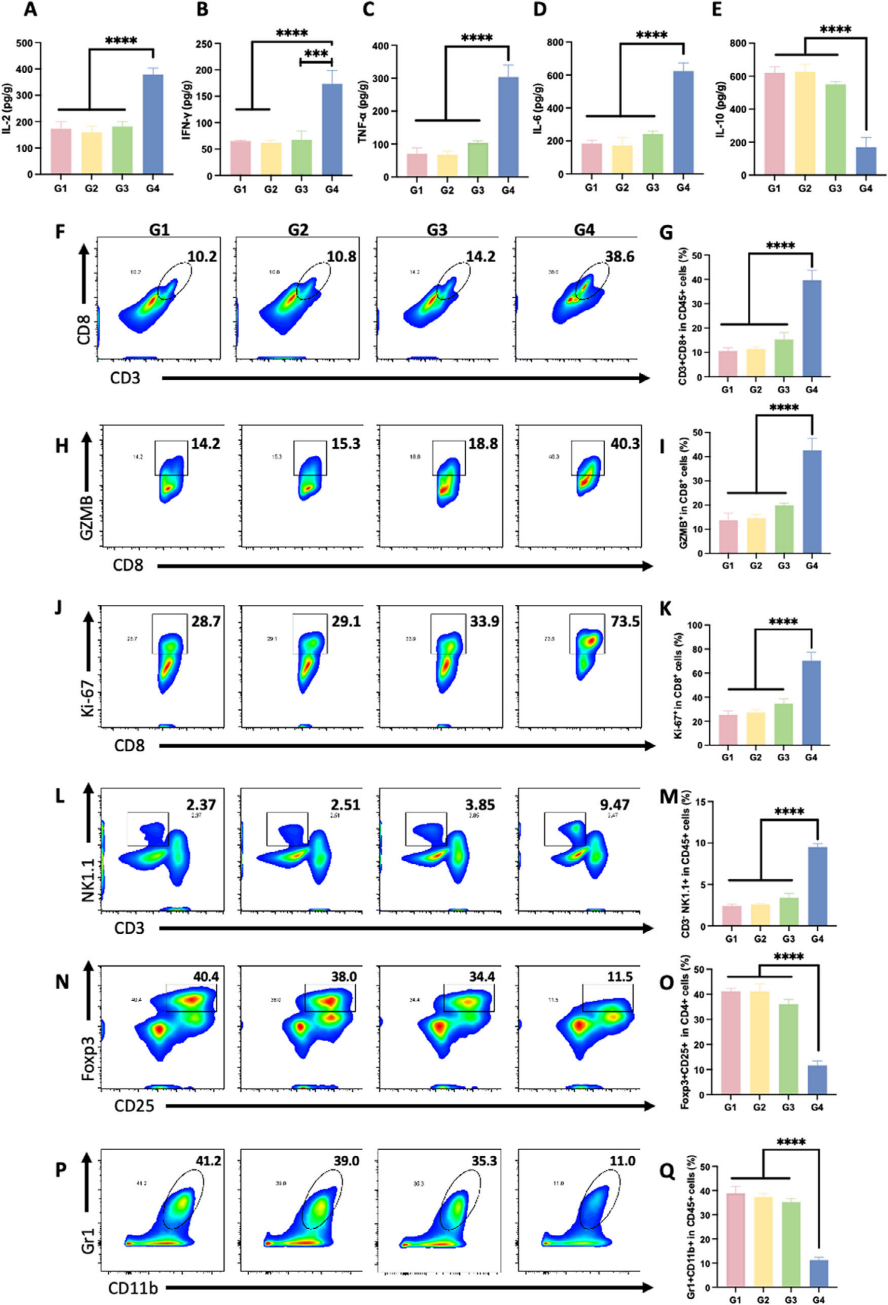
Anti‐tumor immune cell profile after the treatment of LP_11_[*OVA_8_/CpG/LLP2A*]. Concentrations of IL‐2 A), IFN‐γ B), TNF‐α C), IL‐6 D), and IL‐10 E) in plasma on Day 17 for different treatment groups (*n* = 3). ^***^ *P*<0.001, ^*****^ *P*<0.0001 indicates statistical significance compared to G4. One‐way ANOVA with Turkey's posthoc test. Representative flow cytometry scatter plots and histograms of the proportion of CD3+CD8+ F, G), CD8+GZMB+ H,I), CD8+Ki67+ J,K), CD3‐NK1.1+ L,M), CD25+Foxp3+ N,O), and CD11b+Gr1+ P,Q) in the tumor on Day 17 for different treatment groups. (*n* = 3). G1: Saline, G2: Physical mixture of LP_11_, P_3_‐OVA_8_, CpG and LLP2A‐P_4,_ G3: LP_11_[*OVA_8_/CpG*], G4: LP_11_[*OVA_8_/CpG/LLP2A*]. ^*****^ *P*<0.0001 indicates statistical significance compared to G4, one‐way ANOVA with Tukey's posthoc test. The mice were *s.c*. injected with different treatments, each containing an equivalent OVA_8_ concentration of 5 µM, on Days 4, 6, 9, and 12 following tumor inoculation. Tumors and blood were collected when mice were sacrificed on Day 17.

To elucidate the potential enhancement of antitumor immunity following LP_11_[*OVA_8_/CpG/LLP2A*] stimulation, we investigated the immune cell profile in TDLNs. First, the expression of SIINFEKL bound to H‐2Kb on DCs in TDLNs was determined to characterize the antigen presentation efficiency of LP_11_[*OVA_8_/CpG/LLP2A*] to DCs. The data showed that LP_11_[*OVA_8_/CpG/LLP2A*] treatment resulted in superior SIINFEKL expression compared to free OVA_8_ + CpG or LP_11_[*OVA_8_/CpG*] treatment (Figure 4K,L), which can be ascribed to the effective binding of LLP2A to DCs. This result corroborates our finding that LP_11_[*OVA_8_/CpG/LLP2A*] treatment significantly elevated the secretion of antitumoral cytokines (IL‐2, IFN‐γ, and TNF‐α) while decreasing anti‐inflammatory IL‐10 level (**Figure** **5**A–E). In contrast, PM[*LP_11_*/*OVA_8_/CpG/LLP2A*] and LP_11_[*OVA_8_/CpG*] showed insignificant enhancement of these cytokines and less inhibition of IL‐10 secretion. Furthermore, flow cytometric analysis of the immune cell profiles demonstrated that LP_11_[*OVA_8_/CpG/LLP2A*] significantly increased the proportion of CD3+CD8+ T cells, CD8+GZMB+ T cells, and CD8+Ki‐67+ T cells compared to LP_11_[*OVA_8_/CpG*] or PM[*LP_11_*/*OVA_8_/CpG/LLP2A*], indicating expansion of cytotoxic CD8+ T cells (Figure 5F–K). In addition, LP_11_[*OVA_8_/CpG/LLP2A*] was found to outperform other treatments by significantly enhancing the proportion of CD3‐CD49b+NK T cells while decreasing CD25+Foxp3+ regulatory T cells and CD11b+Gr1+ MDSCs in the tumor tissues (Figure 5L–Q). Immunofluorescence analysis of tumor sections further confirmed the expansion of CD3+CD8+ T cells and NK cells caused by LP_11_[*OVA_8_/CpG/LLP2A*] (Figure S15, Supporting Information). The active cytotoxic CD8+T cells after LP_11_[*OVA_8_/CpG/LLP2A*] treatment were found to exert a significantly higher level of IFN‐γ and TNF‐α in the tumor region than those of other treatments (Figure S16, Supporting Information), which would cause an enhanced antitumor effect on tumor regression. Noteworthily, no significant systemic toxicity was observed in any treatment group, as evidenced by normal histology of H&E‐stained major organs (heart, liver, spleen, lung, and kidney) over 17 days of treatments (Figure S17, Supporting Information), confirming the safety of the PVN platform for in vivo applications.

## 3 Conclusion

We have developed a novel, highly versatile, and efficacious PNA‐based vaccine nanosystem (PVN) by taking advantage of the compatibility of peptide and PNA in solid‐phase peptide synthesis. The PNA scaffold allows for convenient, one‐pot self‐assembly of key tumor vaccine components—the tumor antigen OVA_8_ peptide, the adjuvant CpG, and LLP2A (a high‐affinity, high‐specificity peptidomimetic ligand against activated α4β1 integrin expressed on a range of immune cells and melanoma cells)—to form LP_11_[*OVA_8_/CpG/LLP2A*] through Watson‐Crick base pairing. Super‐resolution images revealed the spatial distribution of OVA_8_ within the PVN, and CD spectra of LP_11_[*OVA_8_/CpG/LLP2A*] indicated that PNA bound to complementary oligonucleotides in a parallel orientation. LP_n_[*OVA_8_/CpG/LLP2A*] was found to accumulate in lysosomes, where OVA_8_ was presumably released for antigen presentation.

Among the three LP_n_[*OVA_8_/CpG/LLP2A*] constructs evaluated, LP_11_[*OVA_8_/CpG/LLP2A*] was determined to exhibit the most potent anti‐tumor immune response against B16‐OVA syngeneic tumors. Such a robust antitumor immune response was characterized by enhanced CD8+ T cell activity, NK cell proliferation, and suppression of Treg cells, which could be attributed to the high binding affinity of LLP2A toward activated α4β1 integrin on immune cells in the TDLNs. While the exact reasons for the superior performance of LP_11_[*OVA_8_/CpG/LLP2A*] are not fully established, we can infer that its enhanced antitumor efficacy may lie in its superior stability and optimal surface charge. LP_11_[*OVA_8_/CpG/LLP2A*] is more stable than both LP_10_[*OVA_8_/CpG/LLP2A*] and the rapidly aggregating LP_12_[*OVA_8_/CpG/LLP2A*], which is critical for efficient transport and accumulation in the lymph nodes after *s.c*. injection. Additionally, the zeta potential of LP_11_[*OVA_8_/CpG/LLP2A*] is closer to zero compared to the others, which could reduce electrostatic repulsion with negatively charged cell membranes and facilitate internalization by APCs. Collectively, these characteristics of enhanced stability and optimized surface charge likely contribute to the more effective in vivo delivery and cellular uptake of LP_11_[*OVA_8_/CpG/LLP2A*], ultimately leading to a more robust immune response.

The PVN reported here is robust, modular, non‐toxic, and highly versatile. Through sequence‐specific reannealing, antigenic peptides, immunomodulators, immune cell targeting ligands, and tumor cell targeting ligands can be easily nanoformulated in a one‐pot manner. Although not reported here, we envision that additional immunomodulators (e.g., resiquimod, 5,6‐dimethylxanthenone‐4‐acetic acid), small molecule drugs, immune checkpoint inhibitors, or chemokines tagged with a short PNA sequence can be loaded if desired. As more antigenic peptides become available, one may be able to tailor specific antigenic peptide(s) for specific patients, allowing for precision tumor immunotherapy.

## 4 Experimental Section

### Materials and Animal Protocols

Fmoc‐PNA monomers were purchased from PNA Bio (USA), and Fmoc‐L‐amino acids were from Sigma‐Aldrich (U.S.A). Fmoc‐TentaGel XV RAM (0.28 mmol g^−1^, RAPP Polymer) was from Rapp Polymere (Tübingen, Germany). HOBT, HBTU, Piperidine, 4‐methyl‐morpholine, 4‐methyl‐piperidine, dimethylformamide (DMF), N,N‐diisopropylethylamine (DIEA), N,N′‐diisopropylcarbodiimide (DIC), triisopropylsilane (TIS), m‐Cresol, Trifluoroacetic acid (TFA) were from Sigma–Aldrich. Cyanine 5.5 DBCO and sulfo‐Cyanine 5 DBCO were purchased from Lumiprobe. The CpG (i.e., ODN 1585) used in this study was purchased from Invivogen. Fmoc‐aeea was purchased from Aaron Chemicals. Ultra Centrifugal filter units (Amicon) were bought from Millipore.

### CpG sequence: 5′ ggGGTCAACGTTGAgggggg 3′

Bases in capital letters were phosphodiester and those in lowercase were phosphorothioate.

All experiments were performed following protocols approved by the IACUCs of Ruige Biotechnology (20220905‐001) and University of California, Davis (22533).

### Synthesis of PNA Sequences

The PNA sequences were synthesized by stepwise solid phase synthesis as described previously^[^ ^45^ ^]^ except that the TentaGel XV‐RAM Resin (loading capacity: 0.28 mmol g^−1^, RAPP polymer) was used. The synthesized PNA sequences were purified by RP‐HPLC (Shimadzu, Model: CBM‐20A) and identified by MALDI‐TOF mass spectrometry (MALDI‐TOF‐MS, Bruker).

### PNA Sequences: (N’)‐(C’)

LP_10_: CCCCGTAGCT
LP_11_: CCCCGTATGCA
LP_12_: CCCCTCTATGCA

The peptide sequence listed below was synthesized by using the coupling reagents HOBT and DIC as described in the previous work.^[^ ^46^ ^]^

### Peptide Sequences

OVA_257‐264_ (i.e., OVA_8_): SIINFEKL
LLP2A*‐(aeea)*_2_‐K‐biotin

### PNA‐Peptide Sequences

LLP2A‐P_3_: LLP2A‐(aeea)_2_‐*AGC*
LLP2A‐P_4_: LLP2A‐(aeea)_2_‐*TGCA*
PNA_4_KOVA_8_ (*i.e*., P_4_’OVA_8_): *TAGA*‐K‐SIINFEKL
PNA_3_KOVA_8_ (*i.e*., P_3_’OVA_8_): *TAC*‐K‐SIINFEKL

Note: Bases in italics and capital letters were PNA monomers, and the rest letters denote the L‐amino acids.

### Synthesis of Azide‐aeea‐PNA_n_‐K‐OVA_8_

For the coupling of azide on the N’ terminus of the PNA/peptide sequence, 6 eq of 4‐azidobutyric acid, 6 eq HATU, and 12 eq DIEA were used to react with the amine group of aeea on the resin, and were allowed to react for 2 h at RT.

The dyes, including sulfo‐Cy5‐DBCO and Cy5.5‐DBCO, were coupled to Azido‐aeea‐PNA_n_‐K‐OVA_8_ in DMSO using a 1:1.1 molar ratio of Cy5.5‐DBCO and Azide‐aeea‐PNA_n_‐K‐OVA_8_ via click chemistry. The synthesized dye‐labeled PNA/peptide was purified by RP‐HPLC and identified by MALDI‐TOF mass spectrometry (MALDI‐TOF‐MS).

### Preparation of LP_n_[*OVA_8_/CpG/LLP2A*]

P_n_’OVA_8_, CpG, and LLP2A‐P_n_ and LP_n_ (*n* = 10,11,12) were assembled through a heating‐reannealing process as previously described with some modifications.^[^ ^47^ ^]^ In brief, an excess amount of P_n_’OVA_8_, CpG, and LLP2A‐P_n_ were mixed with LP_n_ (*n* = 10,11,12) at a molar ratio of 6:6:6:1 in 10 mm phosphate (containing 100 mm NaCl, 0.1 mm EDTA at pH 7.0). The mixture was annealed from 65 to 20 °C at the rate of 1 min/0.1 °C. Immediately following this step, centrifugation was performed to remove all the unbound components using a 10 kDa centrifugal filter at 13 000 rpm for 10 min. To quantify the amount of payloads and LP_n_, the PVNs in the centrifuge insert were detached by adding acetonitrile and were centrifuged by a centrifugal filter unit (Cutoff: 5 kD) at 13 000 rpm for 10 min. CpG (MW: 6431) stayed in the insert of the filter unit, while other segments with molecular weight < 3.5 kD were filtered out in the outer centrifuge tube. The concentration of CpG was quantified by NanoDrop Microvolume Spectrophotometer (Thermo Fisher Sci.), while the concentrations of LP_n_, P_n_’OVA_8,_ and LLP2A‐P_n_ were quantified by analytical HPLC (Shimadzu). The ratio of each payload to LP_n_ was calculated as n_payload_/n_LPn_.

### Spatial Distribution of LP_n_[*OVA_8_/CpG/LLP2A*]

For high‐resolution imaging of the LP_n_[*OVA_8_/CpG/LLP2A*], the solutions in all groups containing equivalent OVA_8_(Cy5) at a concentration of 25 µΜ were spined down at 12 000 rpm for 10 min, and the pellets were resuspended with 20 µL fresh dSTORM imaging buffer (ONI, Part A: Part B = 9:1, v/v). A sample droplet was added to a glass slide and covered with a cover slip. The special distribution of P_n_‐OVA_8_(Cy5) was observed under direct stochastic optical reconstruction microscopy (dSTORM), and the images were processed using CODI software.

### CD Spectra Scanning

The samples, including CpG, LP_11_, LP_11_[*CpG*], LP_11_[*OVA_8_*], LP_11_[*LLP2A*], LP_11_[*OVA_8_/CpG*], LP_11_[*CpG/LLP2A*], LP_11_[*OVA_8_/LLP2A*], and LP_11_[*OVA_8_/CpG/LLP2A*], were prepared for CD spectra scanning, each containing equivalent concentrations of CpG, OVA_8_, and/or LP_11_ at 30 µM. The complementary PNA sequences in each group were hybridized in the PBS solution (pH 7.4) without Cl^−^ and were supplemented with 10 mm NaF and 0.1 mm EDTA. The CD spectra of each sample were scanned by a Jasco 715 CD spectrometer with 2 scans on average for each sample.

### Size, Zeta Potential, and Morphology of LP_n_[*OVA_8_/CpG/LLP2A*]

The size and zeta potential of LP_n_[*OVA_8_/CpG/LLP2A*] in H2O were detected by Malvern DLS zetasizer with the equivalent concentration of OVA_8_ or CpG at 5 µΜ. The morphology of LP_n_[*OVA_8_/CpG/LLP2A*] was visualized by transmission electron microscope (TEM, 80 kV FEI Talos 120C).

### Determination of IL‐2 Secretion

The differentiation of BMDC from bone marrow cells was performed as previously described.^[^ ^48^ ^]^ 2 × 10^4^ cells/well BMDCs were suspended in 200 µL medium without adding IL‐2 and IL‐4. BMDCs were treated with LP_n_[*OVA_8_/CpG/LLP2A*] containing 1 or 2 µm of OVA_8_ for 16 h. Each group contains approximately equivalent concentrations of CpG and OVA_8_ based on the data in Table S1 (Supporting Information). After that, the cells were centrifuged at 320 g for 5 min to remove the uninternalized drugs. Then the BMDC cells were resuspended in RPMI medium and were co‐incubated with 10 × 10^4^ cells/well of RF33.70 cells for another 16 h. After that, the medium supernatant was collected. The IL‐2 concentration was measured by IL‐2 Mouse ELISA Kit (Thermo Fisher).

For the blocking experiment, BMDC cells were pre‐incubated with an excess of LLP2A‐P_4_ (100 µm) for 1 h. Then the cells were washed three times with PBS, before the treatment of 1 µm LP_11_[*OVA_8_/CpG/LLP2A*] for 16 h. After that, the cells were washed three times with PBS to get rid of the uninternalized drugs, followed by co‐culturing with RF33.70 cells. The IL‐2 level was quantified by ELISA as mentioned above.

### Antitumor Efficacy Evaluation of LP_n_[*OVA_8_/CpG/LLP2A*]

B16‐OVA‐Luc melanoma cells (3 × 10⁵) were subcutaneously injected into the left flank of mice between the tumor and the inguinal region on Day 1. Tumor‐bearing mice were randomized into six groups and treated subcutaneously on Days 4, 6, 9, and 12 with Saline, PM[*OVA_8_/CpG*], LP_10_[*OVA_8_/CpG/LLP2A*], LP_11_[*OVA_8_/CpG/LLP2A*], or LP_12_[*OVA_8_/CpG/LLP2A*] between the tumor and left inguinal region. Each formulation contained 5 µm LP_n_‐OVA_8_ and ≈5 µm CpG (Table S1, Supporting Information). Tumor growth and body weight were monitored every other day, and tumor volume was calculated as V = 0.5 × L × S^2^. Mice were sacrificed when the tumor volume reached 2000 mm^3^. On Day 20, blood was collected for biochemical and cytokine (IFN‐γ, IL‐6, IL‐12) analyses by ELISA. Tumors and major organs were excised for flow cytometry, and tumor and spleen weights were recorded.

For evaluating LP11‐based incomplete PVNs, B16‐OVA‐Luc tumor models were established similarly and treated with PM[*LP_11_/OVA_8_/CpG/LLP2A*], LP_11_[*OVA_8_*], LP_11_[*OVA_8_/LLP2A*], LP_11_[*OVA_8_/CpG*], or LP_11_[*OVA_8_/CpG/LLP2A*] under the same regimen, with termination on Day 17. Tumor activity was visualized by bioluminescence imaging, survival was recorded, and cytokines (IL‐2, IFN‐γ, IL‐12, IL‐1β, IL‐6, TNF‐α) were quantified by ELISA. Detailed procedures are provided in the Supporting Information.

### Flow Cytometric Analysis of Immune Cell Profiles

Tumor‐infiltrating lymphocytes (TILs) and cells from TDLNs were collected as previously described.^[^ ^49^ ^]^ Freshly isolated cells were resuspended in Flow Cytometry Staining Buffer (PBS with 2% FBS and 0.1% sodium azide) and blocked with Fc‐receptor solution. Cells were stained with fluorochrome‐conjugated antibody panels specific for TIL subsets, washed, and analyzed on a Cytek Aurora flow cytometer. Dead cells were excluded using propidium iodide. Data were processed with FlowJo v10, and positive populations were identified by gating against negative controls. Detailed antibody panels, procedures, and gating strategies are provided in the Supporting Information.

### Histological Analysis of Tumor Tissues

The immunofluorescence staining of tumor tissues was performed as previously described.^[^ ^49^ ^]^

### Statistical Analysis

The experimental data were statistically analyzed using SPSS Statistics and GraphPad Prism Software. A statistically significant difference was reported if *P*< 0.05. Data were reported as the mean ± SD with the sample size indicated in the method section or figure legends. Statistical significance between the groups was determined by the log‐rank Mantel‐Cox test, one‐way, or 2‐way analysis of variance (ANOVA). *P* values less than 0.05 were considered statistically significant.

Methods for 1D proton NMR characterization of CpG ODN 1585, Preparation of ultrathin TEM samples, Quantification of the contact area between BMDC and RF33.70 cells, Differentiation of BMDC, Cell culture for RF33.70 cells, Cellular uptake efficiency of OVA_8_ by BMDCs, Intracellular trafficking of LP_n_[*OVA_8_/CpG/LLP2A*], α4β1 integrin expression and competitive binding assay, Release profile of LP_n_[*OVA_8_/CpG/LLP2A*], Stability of LP_n_[*OVA_8_/CpG/LLP2A*], Biodistribution of PVNs at different formulations, Quantification of blood biochemistry indices are described in the Supporting Information.

### Code Availability

All the codes used for the computational results are available upon request.

## Conflict of Interest

The authors declare no conflict of interest.

## Supporting information



Supporting Information

## Data Availability

The data that support the findings of this study are available from the corresponding author upon reasonable request.
