# Supplementary material for: Designing Programmable Peptide Nucleic Acid‐based Nanovaccines for Anticancer Immune Activation
Source: Small. 2025 Nov 7;21(51):e05605. doi: 10.1002/smll.202505605 (PMC12723328; doi:10.1002/smll.202505605)
Supplement: Supplementary file 1 — Supporting Information [file SMLL-21-e05605-s001.docx]

**Supporting information**

Designing programmable peptide nucleic acid-based nanovaccines for anticancer immune activation

Yanyu Huang, Cuiqing Huang, Sakshi Pandita, Chon Man Ieong, Yongheng Wang, David Wang, Jifeng Chen, Victorio Jauregui-Matos, Alessandra Maria Arabelle Beelen, Ya-Ping Shiau, Shiqi Tang, Junwei Zhao, Qiufang Zong, Menghuan Tang, Zhaoqing Cong, Yuanpei Li, Peter A. Beal, Sheila S. David, Aijun Wang, Duo Wang*, Zeyu Xiao*, Kit S. Lam*

Yanyu Huang, Sakshi Pandita, Yongheng Wang, Ya-Ping Shiau, Shiqi Tang, Junwei Zhao, Qiufang Zong, Menghuan Tang, Zhaoqing Cong, Yuanpei Li, Kit S. Lam*

Department of Biochemistry and Molecular Medicine, University of California, Davis
Sacramento, CA, 95817, USA
E-mail: kslam@ucdavis.edu

Jifeng Chen, Zeyu Xiao*

The Guangzhou Key Laboratory of Molecular and Functional Imaging for Clinical Translation, The First Afﬁliated Hospital of Jinan University, Guangzhou 510632, China

E-mail: zeyuxiao@jnu.edu.cn

Duo Wang*

Center of Interventional Radiology & Vascular Surgery, Department of Radiology, Zhongda Hospital, Medical School, Southeast University, Nanjing 210009, China

Email: wangduo2022@126.com

Cuiqing Huang

Guangdong Women and Children Hospital, Guangzhou 511442, China

Chon Man Ieong

Department of Radiology, Conde de São Januário Hospital, Estrada do Visconde de S. Januário, Macau 999078, Macao SAR, China.

Yongheng Wang, David Wang, Aijun Wang

Department of Biomedical Engineering, University of California, Davis
Sacramento, CA, 95817, USA

Victorio Jauregui-Matos, Alessandra Maria Arabelle Beelen, Peter A. Beal, Sheila S. David

Department of Chemistry, University of California, Davis

Davis, CA, 95616, USA

**Methods**

**1D proton NMR characterization of CpG ODN 1585**

CpG ODN 1585 solutions (0.25 mM or 0.50 mM) in a 9:1 ratio of H_2_O: D_2_O, containing 100 mM KCl and 10 mM potassium phosphate buffer (pH 7.0), were prepared for 1D proton NMR characterization on a 600 MHz Bruker spectrometer. A final volume of 100 μL sample solution was loaded into a coaxial NMR insert (Norell; Item No: NI5CCI-B). Water suppression was performed using a frequency-selective pulse method, and shimming and locking were carried out on the deuterium signal from D₂O. The sample temperature was maintained at 25 °C for the 0.25 mM oligonucleotide solution and at 37 °C for the 0.50 mM oligonucleotide solution. Free induction decays (FIDs) were Fourier-transformed, and the resulting spectra were baseline-corrected. Chemical shifts (δ) are reported in parts per million (ppm).

**Release profile of** **LP_n_[*OVA_8_/CpG/LLP2A*]**

LP_n=10-12_[*OVA_8_(Cy5)/CpG/LLP2A*] at a concentration of 100 μM were prepared as mentioned above. After centrifugation, 16 μL of the stock solution was mixed with 600 μL PBS at pH 7.4, pH 5.5, and pH 4.5 respectively, under mild shaking at 37°C. At the time intervals of 6 h, 24 h, 48 h, and 72 h, 120 μL of the sample was taken out to the centrifugal filter unit (Cutoff: 10 kD) and was filled up to 240 μL with the corresponding PBS solution. The released P_n_’OVA_8_(Cy5) was spun down to the collection tube at a centrifuge speed of 13,000 rpm for 10 min. The release rate of P_n_’OVA_8_(Cy5) (%) = Released P_n_’OVA_8_(Cy5)/Total amount of P_n_’OVA_8_(Cy5) from the prepared PVNs * 100%

**Stability of LP_n_[*OVA_8_/CpG/LLP2A*]**

LP_n=10-12_[*OVA_8_*(Cy5)/*CpG/LLP2A*] at a concentration of 100 μM were prepared as mentioned above. After centrifugation, PVNs were dispersed in PBS at pH 7.4, 5.5, and 4.5, respectively, at a final concentration of 5 μM. The size of PVNs at different pH levels were measured by Malvern DLS zetasizer.

**Preparation of ultrathin TEM samples**

3 ×10^7^ DC cells were harvested after 7 days of differentiation and were separately seeded in three 5-cm dishes with 5 mL RPMI complete medium. DC was treated with 1) PM[*OVA_8_/CpG*], (2) LP_11_[*OVA_8_/CpG*] or (3) LP_11_[*OVA_8_/CpG*/*LLP2A*] for 16 h, each group containing equivalent OVA_8_ at 2 μM. Each group contains approximately equivalent concentration of CpG and OVA_8_ based on the data in Table S1. After that, the DCs were spun down to remove the free nanoparticles in the medium. Then RF33.70 cells at a 5 times higher density were co-cultured with DCs for another 16 h. After that, the cells were harvested and were fixed with 4% formaldehyde. The TEM images of DCs and RF33.70 cells were captured.

**Differentiation of BMDC**

Differentiation of BMDC from bone marrow was performed as described previously.^[1]^ Briefly, C57 BL/6 mice were euthanized and rinsed liberally in ethanol for 5 min. The hindlimbs were severed, and the marrow was flushed out with RPMI‑1640 medium (Gibco, Thermo Fisher). The medium was filtered through a 70‑μm aperture nylon mesh into a 15 ml centrifuge tube in order to remove small pieces of bone and debris. The tube was centrifuged at 300 x g at room temperature for 5 min, and the supernatant was discarded. Red blood cells were collected and lysed with 1 ml Red Blood Cell Lysis buffer (Thermo Fisher) for 10 min on ice to obtain BM‑derived mononuclear cells (BMDMCs). 40 mL RPMI‑1640 medium containing 10% FBS was added to dilute the lysis buffer at room temperature and the mixture was centrifuged at 300 x g at room temperature for 5 min. The cells with a density of 1x10^6^ cells/mL were cultured in 10 mL fresh RPMI‑1640 medium containing 10% FBS, 20 ng/mL recombinant murine GM-CSF (PeproTech, Inc.), and 10 ng/mL recombinant murine interleukin (IL)‑4 (PeproTech, Inc.) in a humidified 5% CO_2_ incubator at 37˚C.

Following 3 days of incubation, the medium containing non-adherent cells was aspirated and discarded. Fresh complete RPMI‑640 medium (10 mL) containing GM‑CSF (20 ng/mL) and IL‑4 (10 ng/mL) was added. On day 6, the medium was collected, centrifuged at 300 × g at room temperature for 5 min, and the cell pellet was resuspended in 10 mL of complete RPMI 1640 medium containing 20 ng/mL GM-CSF and 10 ng/mL IL-4. The resuspended cells were returned to the incubator for 24 h. The BMDCs were ready to use on Day 7.

**Cell culture for RF33.70 cells**

RF33.70 T-cell hybridoma cells were kindly donated by Dr. Kenneth Rock. This cell line was maintained in RPMI 1640 medium supplemented with 10% FBS, 1% Penicillin streptomycin,1% NEAA (non-essential amino acids), 10 mM HEPES, and 2-Mercaptoethanol (1×).

**Quantification of the contact area between BMDC and RF33.70 cells**

The selected cell TEM images were first loaded from a specified path using the Python Imaging Library (PIL) and then displayed with Matplotlib for preliminary visualization (**Figure S7A**). Following this, each image was converted into a NumPy array to enable pixel-level manipulation. A thresholding technique was applied, setting pixels with an intensity value greater than 210 to 255 (white) and those below 0 (black), effectively binarizing the image (**Figure S7B**). To reduce noise and fill small gaps, a median filter with a size of 7 was applied, followed by a maximum filter. A minimum filter was subsequently used to refine the image by contracting regions that had been overly expanded (**Figures S7C, S7D**). The image was then cropped to a specific region of interest, focusing on the array slice from columns 110 to 680. Finally, the processed image was reverted to grayscale, and the number of white pixels per row was calculated and recorded for further analysis. Subsequently, calculations were performed using the dplyr package in R. Stacked bar charts were then generated using ggplot2.

**α4β1 integrin expression and competitive binding assay**

To verify the binding of LLP2A with α4β1 integrin, six different cell types were used: RF 33.70, BMDCs, CD3+ T cells, CD4+ T cells, CD8+T cells, and B16 OVA cells. CD3+ T cells, CD4+ T cells, and CD8+ T cells were isolated from splenocytes of C57BL/6 mice using T cell isolation kits (R&D Systems, cat numbers:  MAGM201, MAGM205, MAGM203) according to the manufacturer’s instructions. For each cell type, 1 × 10^6^ cells were incubated with LLP2A*-(aeea)*_2_-K-biotin (1 μM) for 30 min at 4 °C in FACS buffer (1% FBS in PBS) containing 1 mM Mn^2+^ ions. Following incubation, the cells were thoroughly washed to remove unbound peptides and subsequently incubated with Streptavidin-PE (R&D Systems) for 30 min. To assess the expression of α4β1 integrin, the cells were stained with mouse-reactive monoclonal antibodies for α4 integrin (CD49d-FITC; BioLegend 103606) and β1 integrin (CD29-AF647; BioLegend 102213). Fluorescence was measured for 250,000 cells using a BD LSR Fortessa Flow Cytometer.

For the blocking experiment, BMDC cells or RF33.70 cells were pre-incubated with an excess of LLP2A (100 μM) for 1 h. Then the cells were washed three times in FACS buffer (1% FBS in PBS), prior to the treatment of LLP2A*-(aeea)*_2_-K-biotin and quantification of α4β1 integrin expression as mentioned above.

**Cellular uptake efficiency of OVA_8_ by BMDCs**

BMDCs after 7 days of differentiation were seeded in 12-well plates at 5 × 10^5^ cells per well. Physical mixture of OVA_8_(Cy5) and CpG, LP_11_[*OVA_8_/CpG*], LP_11_[*OVA_8_(Cy5)/CpG/LLP2A*], LP_10_[*OVA_8_(Cy5)/CpG/LLP2A*], and LP_12_[*OVA_8_(Cy5)/CpG/LLP2A*] were added to the cells containing 2 μΜ OVA_8_(Cy5) and approximately 2 μΜ CpG and incubated for 2 and 8 h, respectively. After that, the cells were collected and rinsed twice with PBS, followed by cell lysis. The uptake efficiency of OVA_8_(Cy5) in BMDCs was quantitatively measured with the excitation and emission wavelength of Cy5 set at 645 nm and 685 nm using a microplate reader (SpectraMax iD5, Molecular Devices). Uptake efficiency was calculated as the percentage of fluorescent intensity of Cy5 in cells versus the original fluorescence intensity in cell medium.

**Intracellular trafficking of LP_n_[*OVA_8_/CpG/LLP2A*]**

To visualize the cellular localization of LP_n_[*OVA_8_/CpG/LLP2A*], P_n_’OVA_8_ (Cy5) was substituted for P_n_’OVA_8_ during the preparation of PVNs as mentioned above. PM[*OVA_8_*(Cy5)*/CpG*], LP_10_[*OVA_8_*(Cy5)*/CpG/LLP2A*], LP_11_[*OVA_8_*(Cy5)*/CpG/LLP2A*], and LP_12_[*OVA_8_*(Cy5)*/CpG/LLP2A*] were incubated with BMDCs for 8 h in a 96-well plate at a density of 4 × 10⁴ cells per well. After 8 h of incubation, the BMDCs were spun down to remove the free drugs in the medium, and then lysotracker green and DAPI were stained with BMDCs to label lysosomes and nucleus. The Cy5 fluorescence in the cells was visualized by using a confocal microscope (LSM800, Zeiss).

**Biodistribution of PVNs at different formulations**

The biodistribution of PVNs was performed in B16-OVA-bearing mice. B16-OVA cells were inoculated on the left flank of mice. PM[*OVA_8_(Cy5.5) + CpG*], LP_11_[*OVA_8_(Cy5.5)/CpG*] and LP_11_[*OVA_8_(Cy5.5)/CpG/LLP2A*] containing 20 μM of *OVA_8_(*Cy5.5) was s.c. injected between the tumor and the left inguen. The whole-body imaging was performed at 24 h, 72 h, and 120 h post-injection with the excitation and emission wavelength set at 710 nm, 740 nm, respectively (n=3). At the time points of 72 h and 120 h post-injection, mice were sacrificed, and the organs, including LN, spleen, tumor, liver, lung, heart, and kidney, were dissected for *ex vivo* imaging. After that, the organs were homogenized with lysis buffer, and the fluorescence of OVA_8_(Cy5.5) was quantified by using a microplate reader (SpectraMax iD5, Molecular Devices).

**Antitumor ability evaluation of LP_n_[*OVA_8_/CpG/LLP2A*]**

3×10^5^ B16-OVA-Luc melanoma cells were subcutaneously injected into the left flank of mice on Day 1. The mice were randomly divided into six groups. On Day 4, Day 6, Day 9, and Day 12, the tumor-bearing mice were subcutaneously injected with different groups, i.e., (1) Saline, (2) PM[*OVA_8_/CpG*], (3) LP_10_[*OVA_8_/CpG/LLP2A*], (4) LP_11_[*OVA_8_/CpG/LLP2A*], (5) LP_12_[*OVA_8_/CpG/LLP2A*] between the left inguen and the tumor site. Each group contains an equivalent concentration of 5 μΜ LP_n_-OVA_8_ and approximately 5 μΜ CpG based on the data in Table S1. The tumor size and body weight were monitored every other day after tumor inoculation using calipers in three dimensions. The tumor volumes were calculated using the formula V = 0.5 × L × S^2^, where L and S are the larger and smaller diameters, respectively. Mice were sacrificed when the size of the tumor reached 2,000 mm^3^. On Day 20, the blood of each mouse was collected before euthanization for the measurement of blood biochemistry index. The tumors and the major organs were dissected and cryopreserved for flow analysis. The tumor weight and spleen weight were measured. The cytokine levels, including IFN-γ, IL-6, and IL-12, in the plasma were detected by ELISA at the end of treatments. To calculate the Relative Tumor Volume (RTV), the tumor volume on a given measurement day (V_t_​) is divided by the initial tumor volume (V_0_​), as expressed by the formula RTV = V_t_ ​/ V_0_​.

To assess the antitumor efficacy of LP_11_-based incomplete PVNs, including PM[*LP_11_/OVA_8_/CpG/ LLP2A*], LP_11_[*OVA_8_*], LP_11_[*OVA_8_/LLP2A*], LP_11_[*OVA_8_/CpG*], LP_11_[*OVA_8_/CpG/LLP2A*], B16-OVA-Luc-bearing -bearing tumor model was established as mentioned above. The dosing regimen and analysis process were the same as mentioned above, but the termination day was moved to Day 17.

At the end of the experiment, the tumor activity in B16-OVA-Luc-bearing mice after all treatments was visualized by using bioluminescence imaging. Additionally, the survival rate of each treatment group was recorded. The cytokine levels, including IL-2, IFN-γ, IL-12, IL-1β, IL-6, and TNF-α in the plasma were detected by ELISA at the end of treatments.

**Flow cytometric analysis of immune cell profiles**

Tumor-infiltrating lymphocytes (TIL) in the tumor or TDLN were collected as in previous protocol.^[2]^ Freshly isolated cells, including tumor cells, LNs, and DCs, were resuspended in Flow Cytometry Staining Buffer (PBS supplemented with 2% FBS and 0.1% Sodium Azide) to a concentration of 1 x 10^7^ cells/mL. To prevent non-specific antibody binding, cells were incubated with an Fc-receptor blocking solution for 10 minutes at room temperature. A pre-titrated cocktail of fluorochrome-conjugated antibodies (listed below) was then added, and the cells were incubated for 30 minutes at 4°C in the dark. Following incubation, cells were washed twice with staining buffer and then resuspended for analysis. Immediately prior to data acquisition, a viability dye, i.e., Propidium Iodide (PI), was added to exclude dead cells. Data were acquired on a flow cytometer (Cytek Aurora), and analysis was performed using FlowJo software (v10, Becton, Dickinson and Company). Positive cell populations were determined by gating against the negative control to distinguish specific fluorescence from autofluorescence. After gating on singlets and live cells, immune populations were identified based on the expression of canonical markers.

For preparing antibody staining cocktails, TILs in lymph nodes were stained with CD45-APC-Cy7, L/D-FITC, CD3-PerCP-Cy5.5, CD8-PE-Cy7, CD11c-BV421, DC40-APC, CD80-PE, CD86-BV510. CD8+T cells in tumors were stained with CD45-APC-Cy7, L/D-FITC, CD3-PerCP-Cy5.5, CD8-BV510, Ki67-BV421, Granzyme-PE, TNF-α-ΑPC, IFN-γ-PE-Cy7. MDSCs in tumors were stained with CD45-APC-Cy7, L/D-FITC, CD11b-PE, Gr-1-BV42. NK cells and Treg cells in tumors were stained with CD45-APC-Cy7, L/D-FITC, CD3-PerCP-Cy5.5, NK1.1-PE, CD4-BV510, CD25-PE-Cy7, and FoxP3-APC. The maturation of DC and antigen presentation efficiency in TDLN was detected after 3 days of treatment. The DCs were stained with CD11c BV421 and Hk2b-APC (25-D1.16). The phenotype of TIL was analyzed by FLOWJO 10.7.1 software.

**Gating strategy:**

**
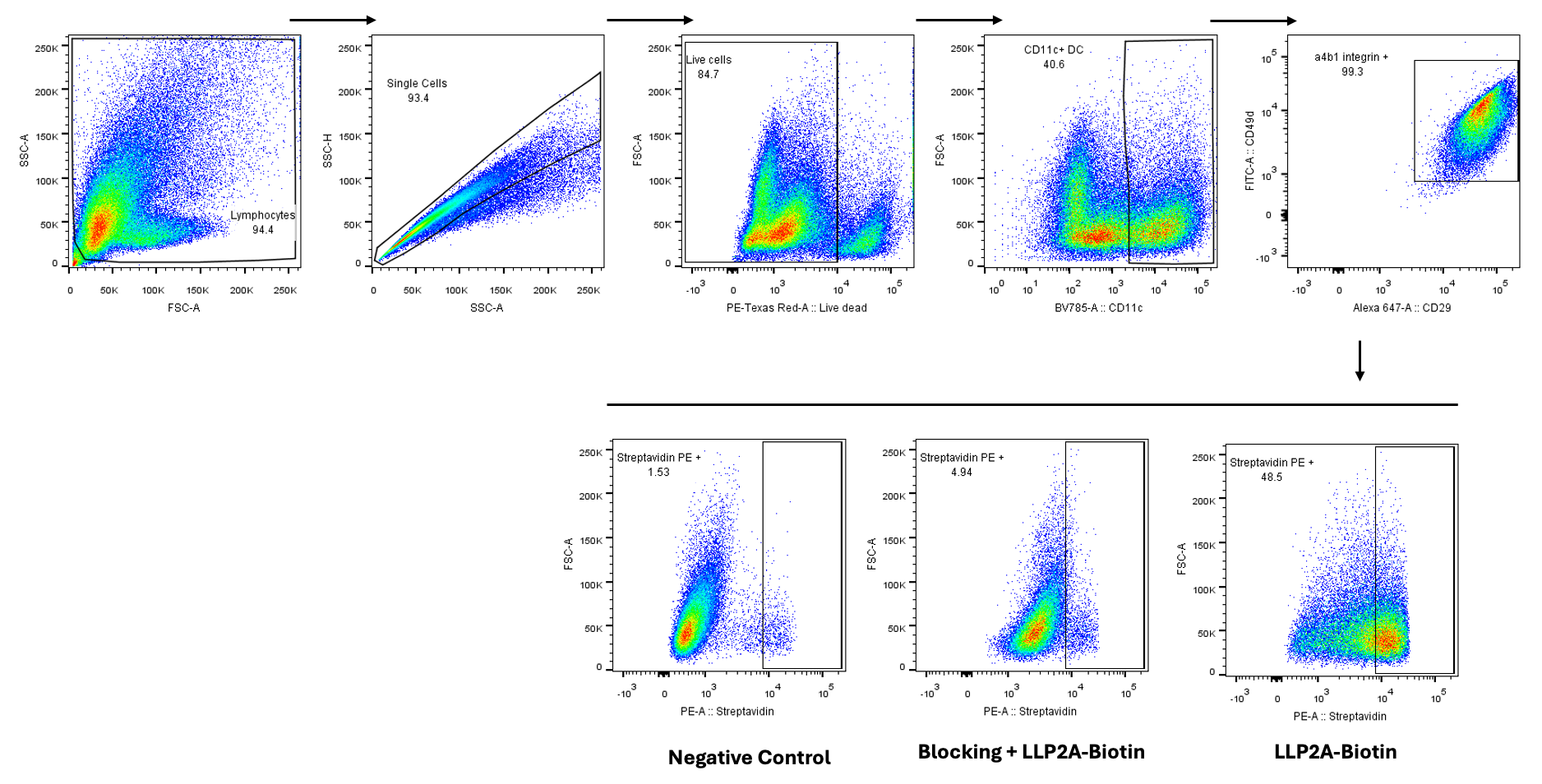
**

**
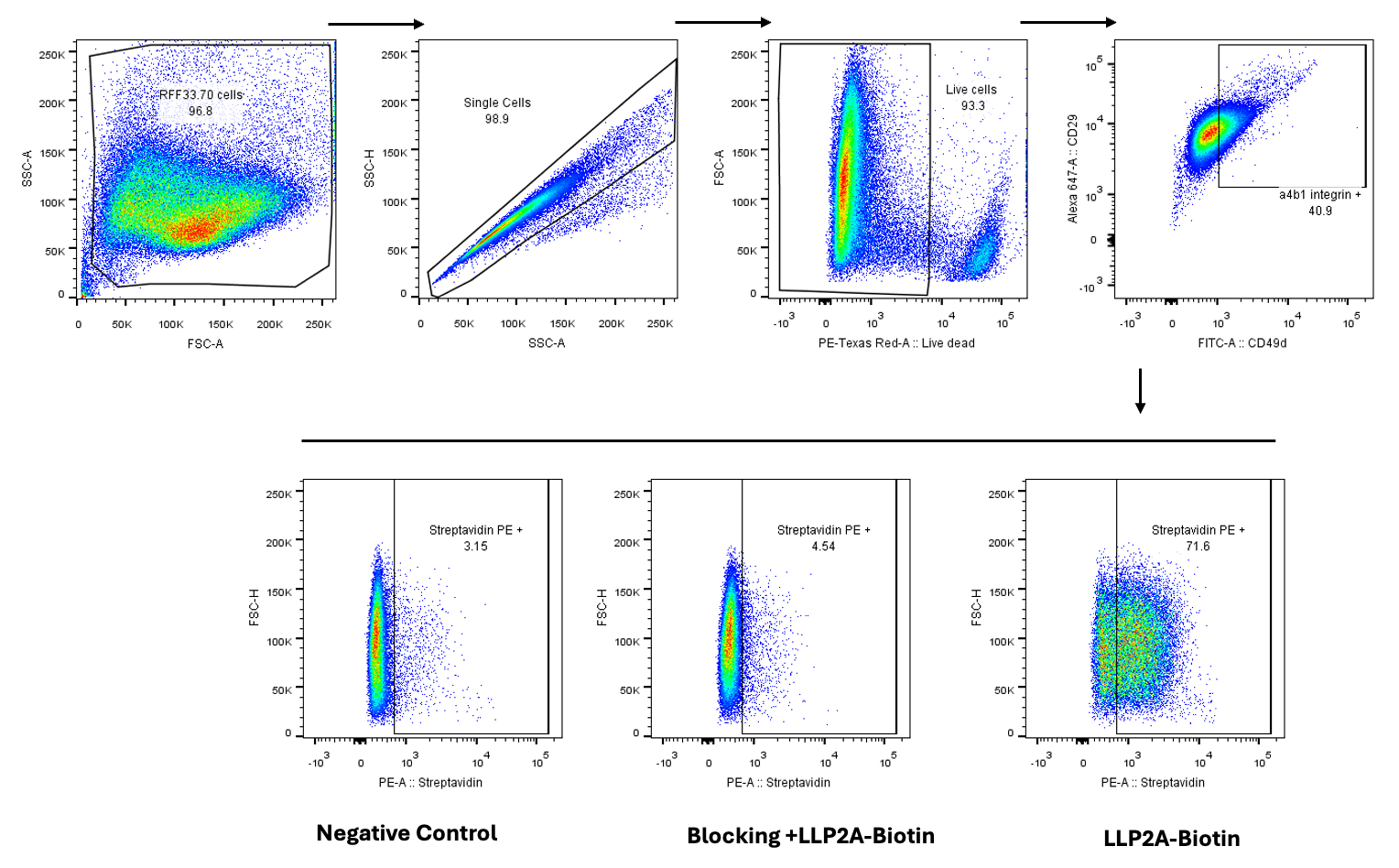
**

**
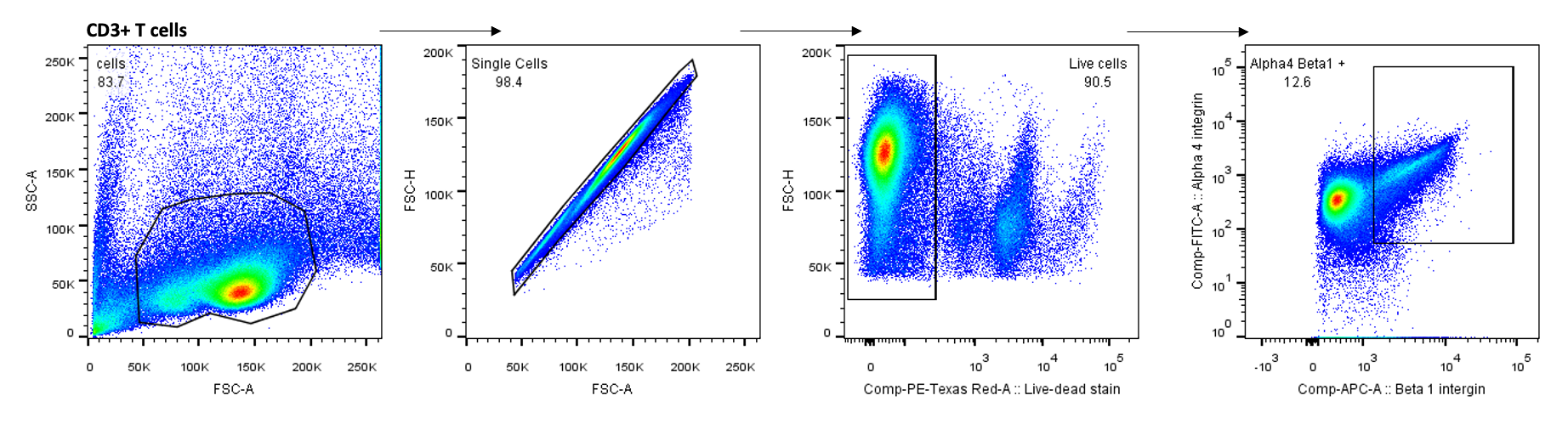
**

**
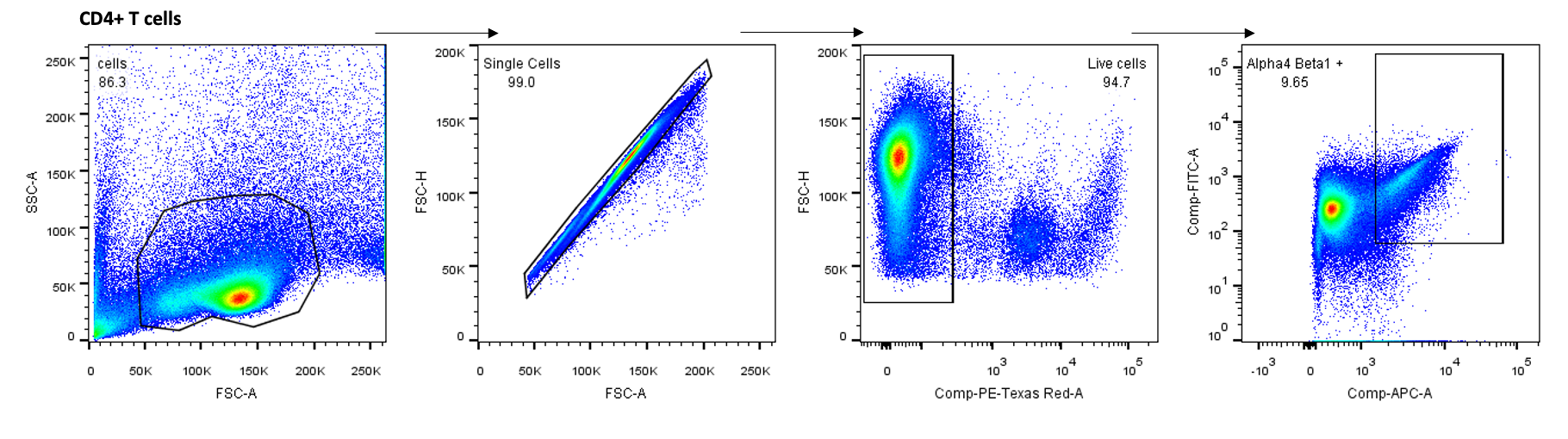
**

**
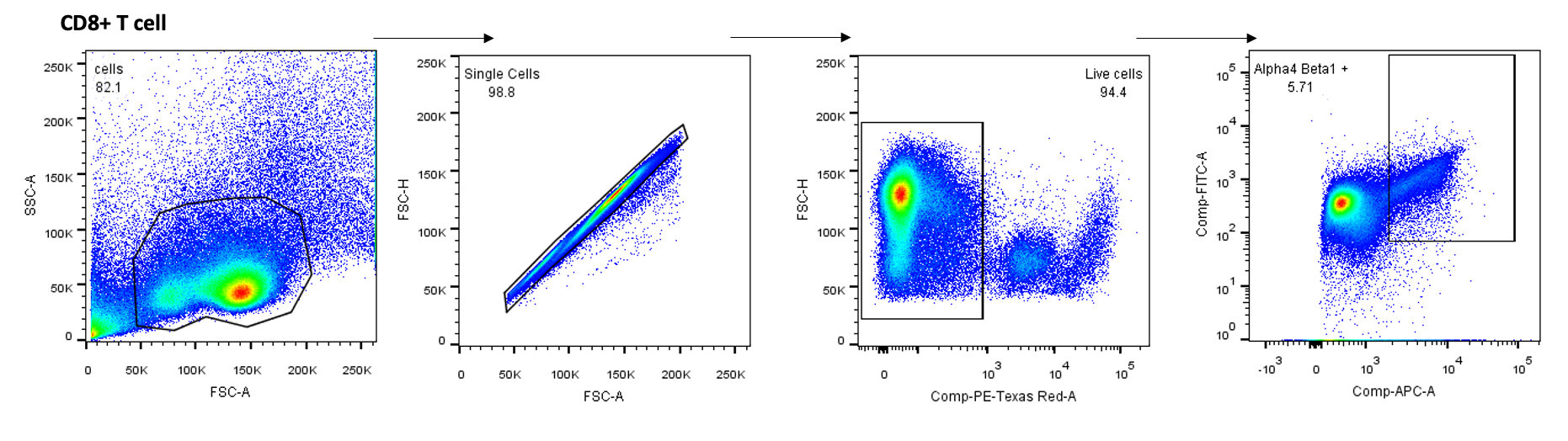
**

**Histological analysis of tumor tissues.**

The immunofluorescence staining of tumor tissues was done as we previously described.^[3]^

**Quantification of blood biochemistry indices**

To evaluate the biosafety of LP_n=10,11,12_[*OVA_8_/CpG/LLP2A*] *in vivo*, C57BL/6 mice were intravenously injected with LP_n_[*OVA_8_/CpG/LLP2A*] containing 5 μM OVA_8_ and approximately 5 μM CpG as detected in Table S1. After 30 days, the blood samples were collected and centrifuged at 3000 rpm to obtain the serum. Biochemical indices in serum including white blood cell (WBC), red blood cell (RBC), platelet (PLT), alanine aminotransferase (ALT), aspartate aminotransferase (AST), albumin (ALB), blood urea nitrogen (BUN), creatinine (CR), and uric acid (UA) were determined by an automatic biochemical analyzer.

**Table S1.** Ratio of payloads to LP_n_ in PVNs

| **PVNs** | **Ratio of payloads to LP_n_** | | |
| --- | --- | --- | --- |
|  | **CpG: LP_n_** | **P_n_-OVA_8_: LP_n_** | **LLP2A-P_n_: LP_n_** |
| LP_10_[*OVA_8_/CpG/LLP2A*] | 1.21: 1 | 1.00: 1 | 1.28: 1 |
| LP_11_[*OVA_8_/CpG/LLP2A*] | 0.90: 1 | 1.17: 1 | 1.05: 1 |
| LP_12_[*OVA_8_/CpG/LLP2A*] | 0.95: 1 | 1.20: 1 | 1.28: 1 |


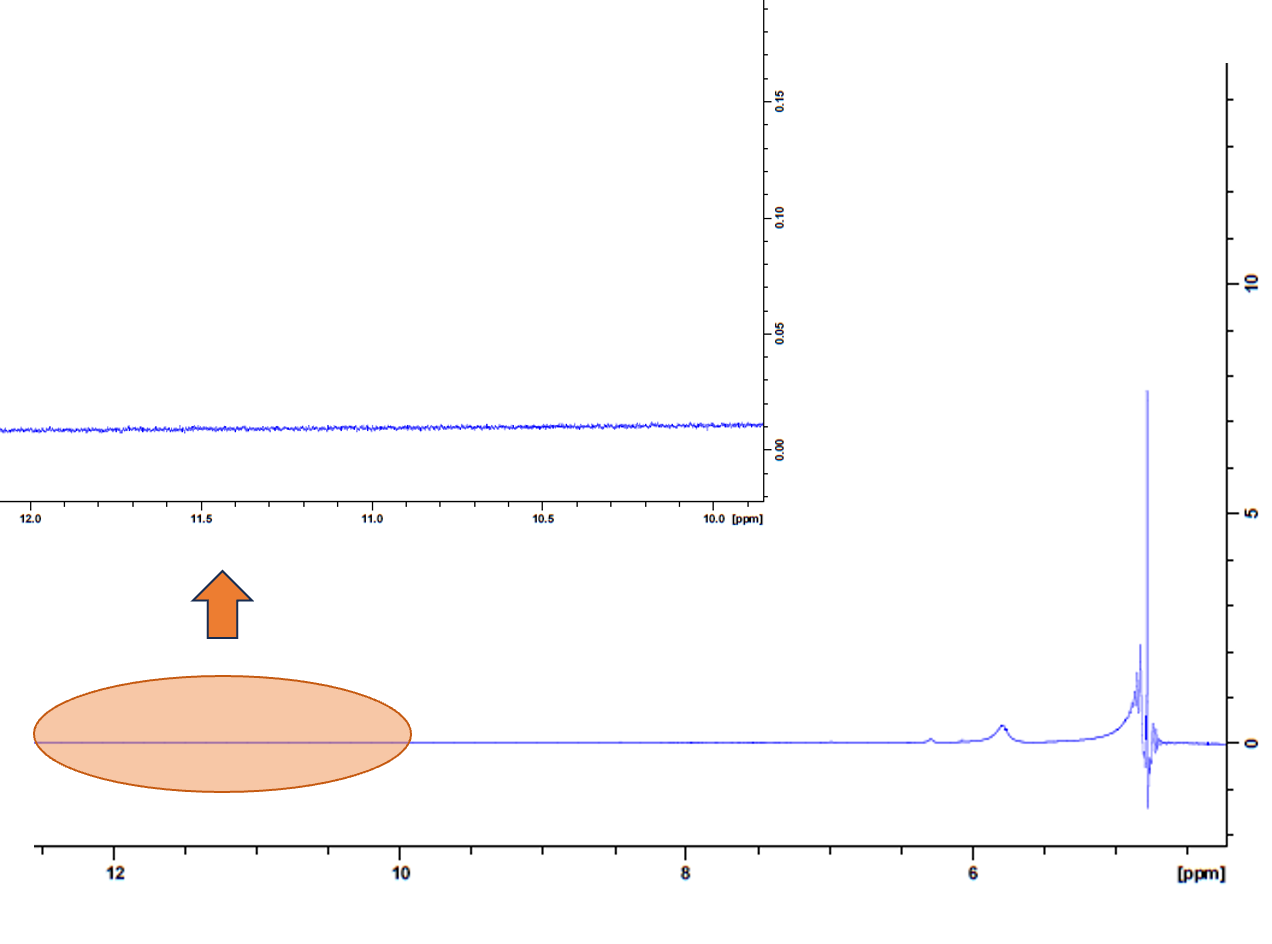


**Figure S1.** The 1D 1H NMR spectrum of CpG (0.5 mM) at 25°C in 100 mM NaCl, pH 7.0.


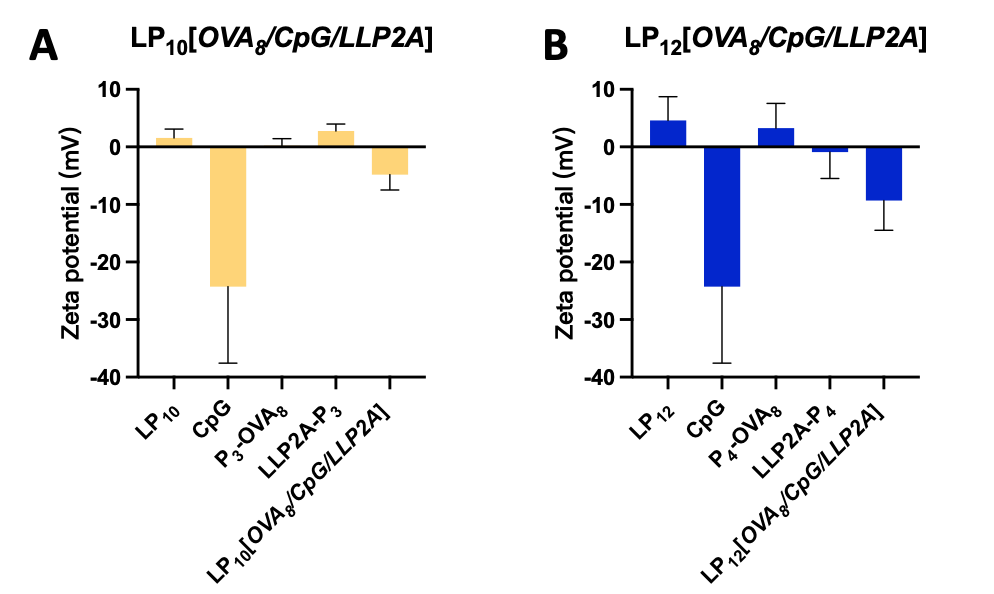


**Figure S2.** Zeta potential values of LP_10_[*OVA_8_/CpG/LLP2A*] and LP_12_[*OVA_8_/CpG/LLP2A*] and the single components in H_2_O detected by DLS. Data were presented as mean ± SD (n=3).

**Figure S3.** Excess LLP2A-P4 competes with Biotinylated-LLP2A for binding on BMDCs (A) and RF33.70 cells (B). BMDCs and RF33.70 cells were pre-activated with 1 mΜ Mn^2+^ before incubation with biotinylated LLP2A and Streptavidin-PE to activate α4β1 integrins.

**Figure S4.** IL-2 level of the co-culture of RF33.70 cells and activated BMDCs for 16 h. Before incubation with RF33.70 cells, BMDCs were blocked with 100 μM of LLP2A-P_4_ for 1 h, and then treated with 1 μM LP_11_[*OVA_8_/CpG/LLP2A*] for 16 h. **P*<0.05, one-way ANOVA with Tukey's posthoc test. Data presented as mean ± SD (n=3).

**
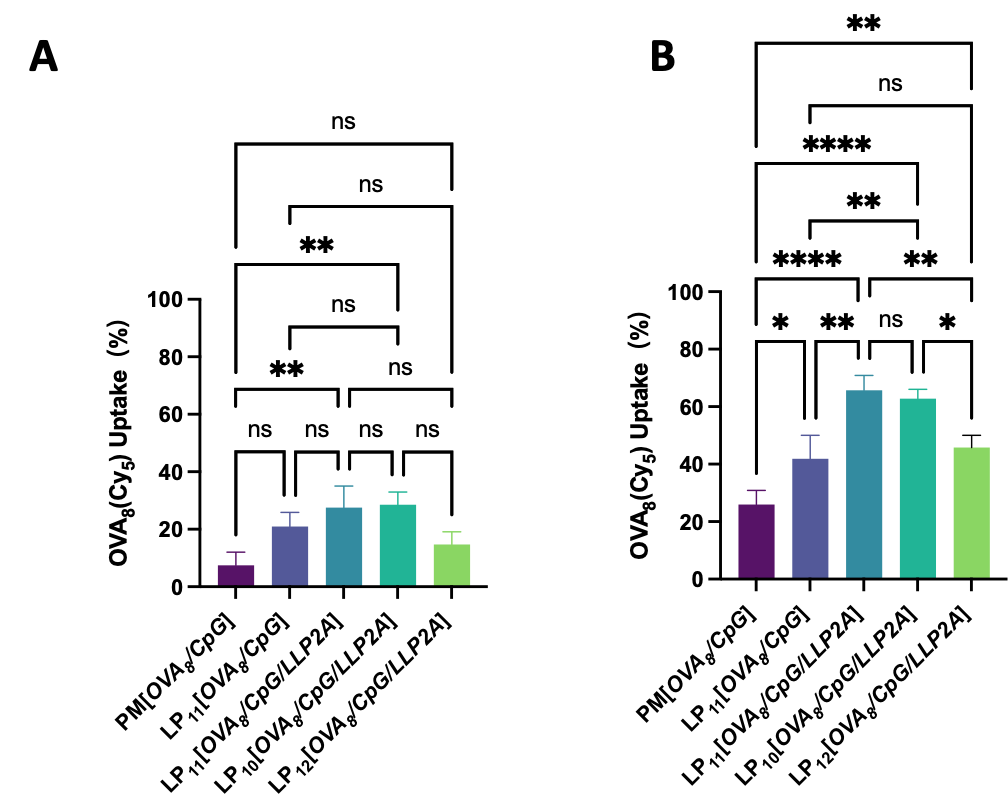
**

**Figure S5.** OVA_8_(Cy5) uptake efficiency by BMDCs after treatments with PM[*OVA_8_(Cy5)/CpG*], LP_11_[*OVA_8_(Cy5)/CpG*], LP_11_[*OVA_8_(Cy5)/CpG/LLP2A*], LP_10_[*OVA_8_(Cy5)/CpG/LLP2A*] or LP_12_[*OVA_8_ (Cy5)/CpG/LLP2A*] for 2 h (A) and 8 h (B), respectively. “ns” denotes “not significant”, * *P*<0.05, ** *P*<0.01, ****P*<0.001, one-way ANOVA with Tukey's posthoc test. Data were presented as mean ± SD (n=3).


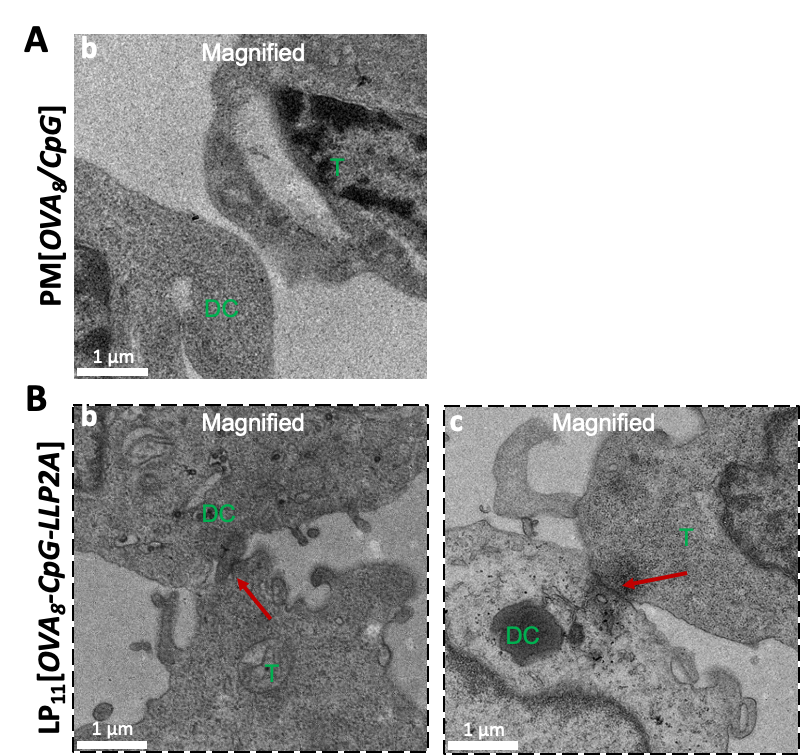


**Figure S6**. (A) Enlarged TEM image of *Zone b* from the TEM image of the physical mixture of *OVA_8_* and *CpG* in Figure 2F. (B) Enlarged TEM image of *Zones b,c* from the TEM image of Group LP_11_[*OVA_8_/CpG/LLP2A*] in Figure 2F. Scale bar = 1 μm. The red arrows indicated the close-contact zone between DC and RF33.70 cells.


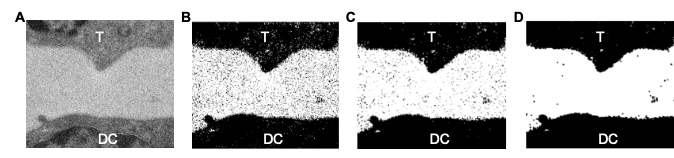


**Figure S7. Procedure for image processing and quantification of the distance between BMDCs and RF33.70 T-cell hybridoma**: (A) Crop image of the interacting region between RF33.70 cells and BMDCs. (B) Adjust the threshold of the image. (C) Apply denoising techniques. (D) Fill in the holes in the image.

**
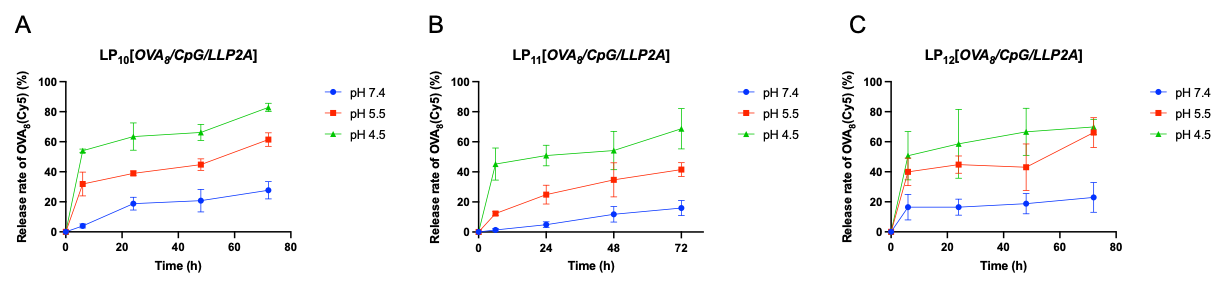
**

**Figure S8.** Release rate of OVA_8_(Cy5) from (A) LP_10_[*OVA_8_(Cy5)/CpG/LLP2A*], (B) LP_11_[*OVA_8_(Cy5)/CpG/LLP2A*], and (C) LP_12_[*OVA_8_(Cy5)/CpG/LLP2A*] in PBS at pH 7.4, pH 5.5, and pH 4.5, respectively. Data presented as mean ± SD (n=3).


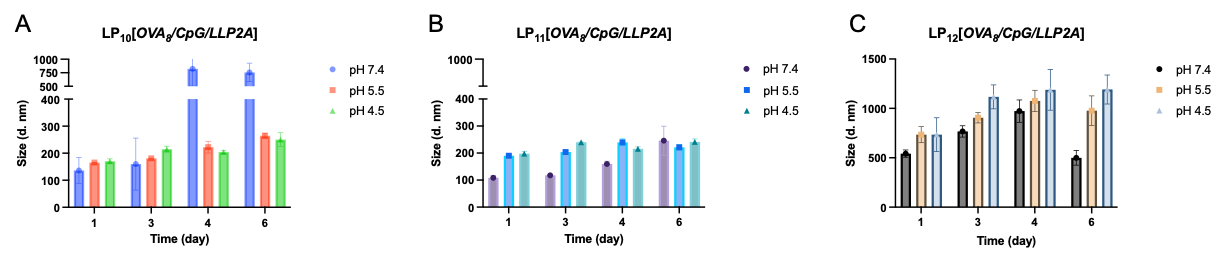


**Figure S9**. Size changes of (A) LP_10_[*OVA_8_/CpG/LLP2A*], (B) LP_11_[*OVA_8_/CpG/LLP2A*], and (C) LP_12_[*OVA_8_/CpG/LLP2A*] in PBS at pH 7.4, pH 5.5, and pH 4.5, respectively. Data presented as mean ± SD (n=3).

**Figure S10. Bodyweight of B16-OVA-bearing mice after various treatments for 20 days.** The mice received subcutaneous injections of various treatments, each containing an equivalent concentration of OVA_8_ at 5 μΜ, on Days 4, 6, 9, and 12 after tumor inoculation (n=6). Treatment groups: Saline, Physical mixture of *OVA_8_* and CpG, LP_10_[*OVA_8_/CpG/LLP2A*], LP_11_[*OVA_8_/CpG/LLP2A*], LP_12_[*OVA_8_/CpG/LLP2A*]. “ns” denotes no statistical significance among different treatment groups.

**Figure S11.** Biochemical indices including (A) WBC, (B) RBC, (C) PLT, (D) ALT, (E) AST, (F) ALB, (G) BUN, (H) CR, and (I) UA in healthy mice after a single intravenous injection of LP_n_[*OVA_8_/CpG/LLP2A*], each containing an equivalent OVA_8_ concentration of 5 μM (n=3). Blood was drawn before the mice were sacrificed on Day 20. G1: Saline, G2: Physical mixture of OVA_8_ and CpG, G3: LP_10_[*OVA_8_/CpG/LLP2A*], G4: LP_11_[*OVA_8_/CpG/LLP2A*], G5: LP_12_[*OVA_8_/CpG/LLP2A*].


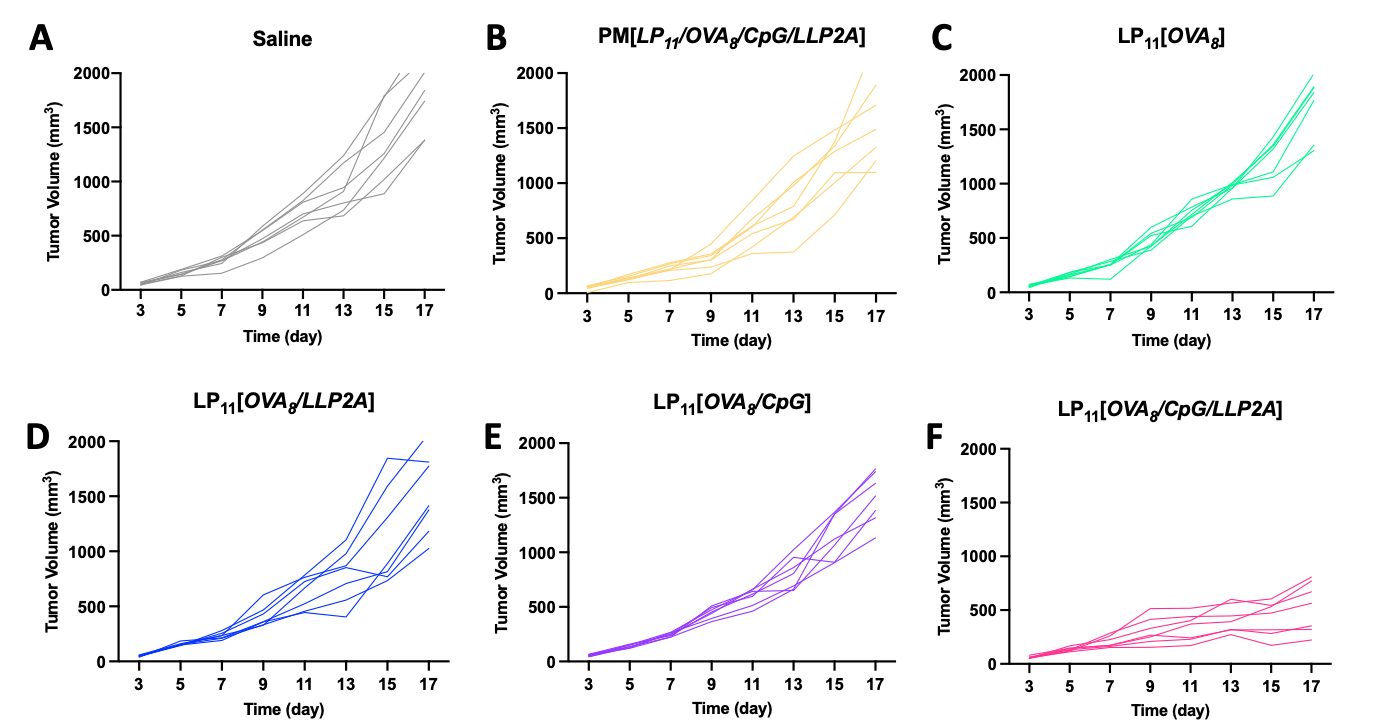


**Figure S12.** Tumor growth curves of B16-OVA-bearing mice after the treatment of (A) saline, (B)Physical mixture of LP_11_, P_3_-OVA_8_, CpG and LLP2A-P_4_, (C) LP_11_[*OVA_8_*], (D) LP_11_[*OVA_8_/LLP2A*], (E) LP_11_[*OVA_8_/CpG*] and (F) LP_11_[*OVA_8_/CpG/LLP2A*] for 17 days (n=7). The mice were *s.c.* injected with different treatments, each containing an equivalent OVA_8_ concentration of 5 μM, on Days 4, 6, 9, and 12 post-tumor inoculation.

**
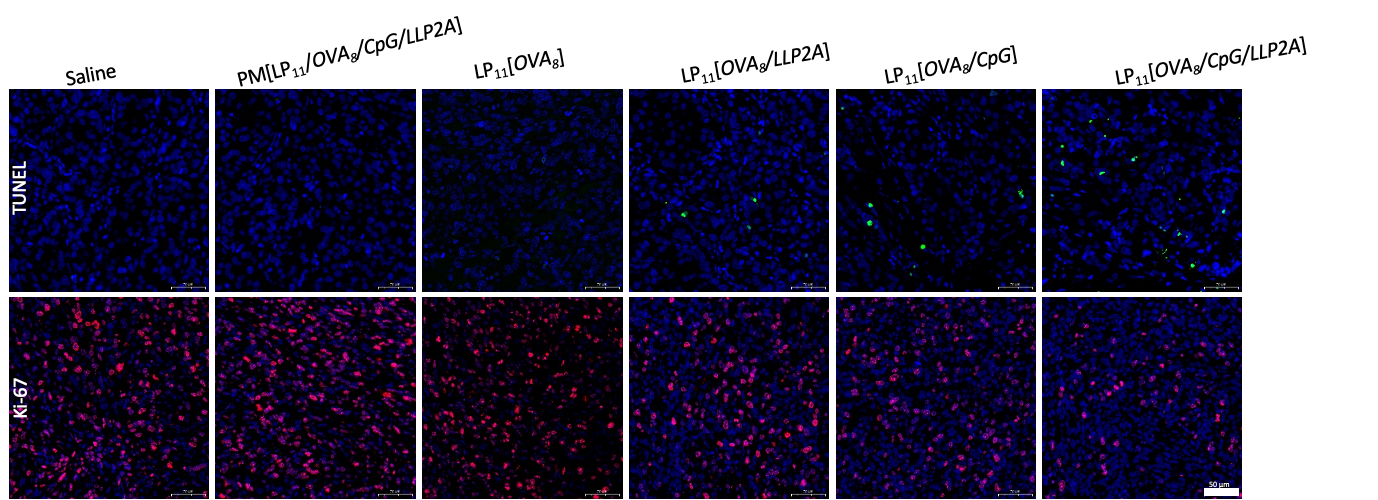
**

**Figure S13.** Immunofluorescent signals of TUNEL and Ki67 in tumor sections after various treatments, scale bar= 50 μm. The mice were *s.c.* injected with different treatments, each containing an equivalent OVA_8_ concentration of 5 μM, on Days 4, 6, 9, and 12 post-tumor inoculation. Tumor tissues were sectioned when mice were sacrificed on Day 17.


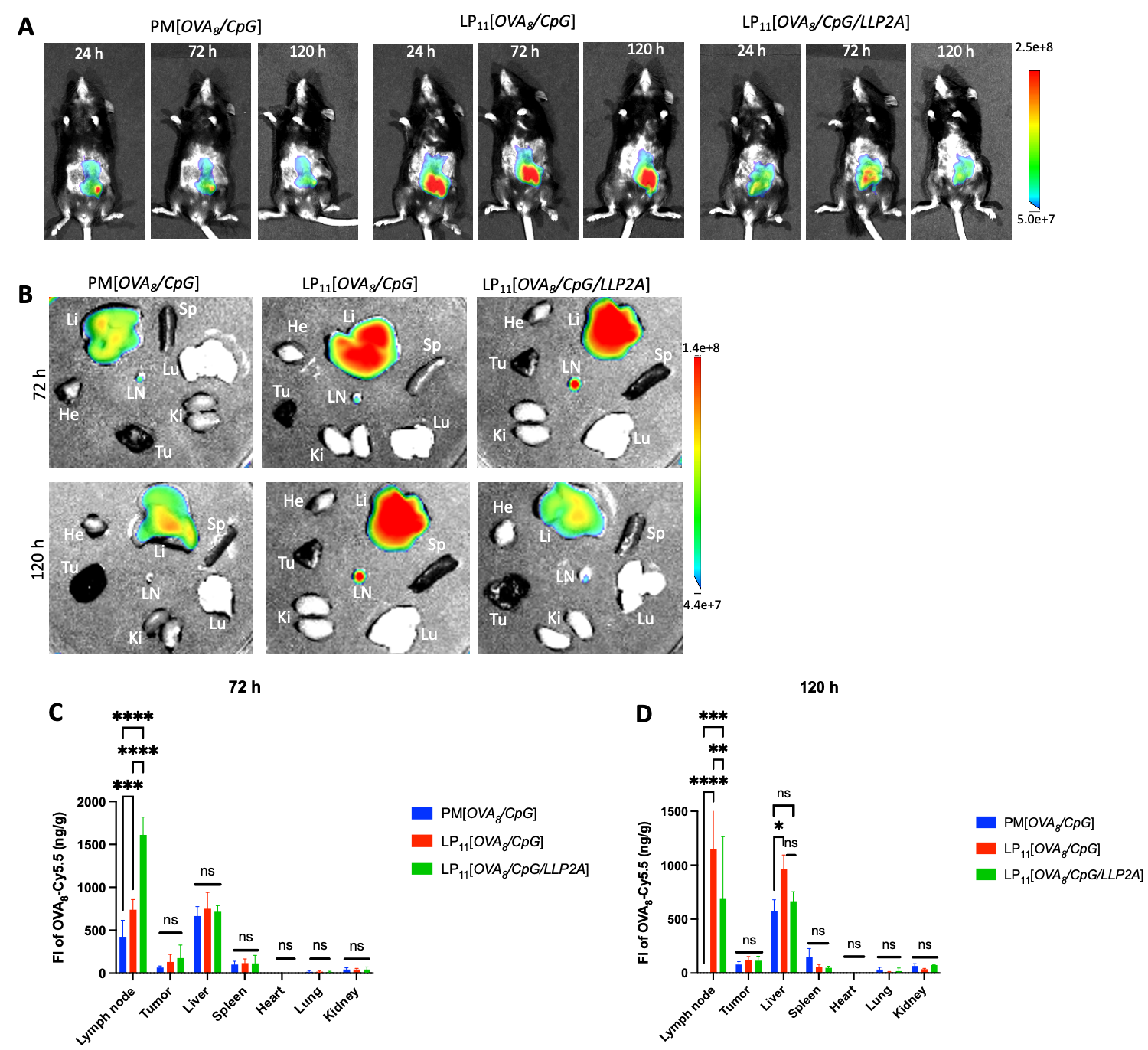


**Figure S14.** (A) Biodistribution of PM[*OVA_8_/CpG*], LP_11_[*OVA_8_/CpG*] and LP_11_[*OVA_8_/CpG/LLP2A*] in B16-bearing tumor model within 5 days of observation (n=3). Each formulation contains an equivalent concentration of 20 μΜ of OVA_8_(Cy5.5). (B) Ex vivo imaging of organs, including the heart, liver, spleen, lung, kidney, lymph node, and tumor, at 72 h and 120 h post-injection of different treatments. Quantification of Cy5.5 fluorescence in lymph node, tumor, liver, spleen, heart, lung, and kidney at 72 h (C) and 120 h post-injection of different treatments (D). Data presented as mean ± SD (n=3). “ns” denotes not significant, ***P*<0.01, *** *P*<0.001, *****P*<0.0001, two-way ANOVA with Tukey's posthoc test.


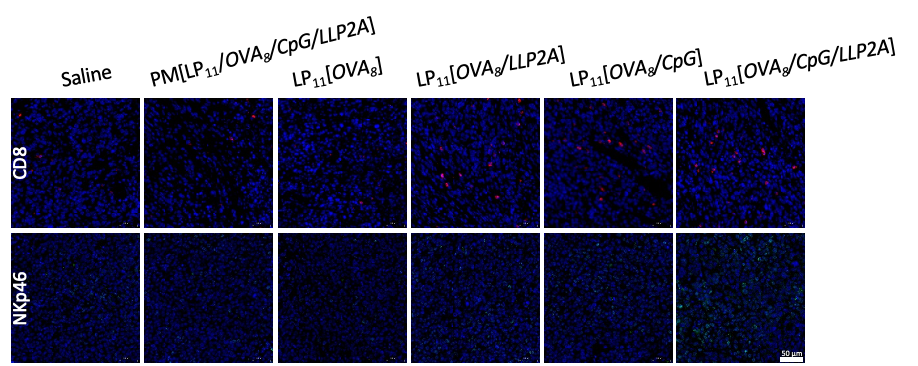


**Figure S15.** Immunofluorescent signals of CD8 and NKp46 in tumor sections after various treatments, scale bar= 50 μm. The mice were *s.c.* injected with different treatments, each containing an equivalent OVA_8_ concentration of 5 μM, on Days 4, 6, 9, and 12 post-tumor inoculation. Tumor tissues were sectioned when the mice were sacrificed on Day 17.

**
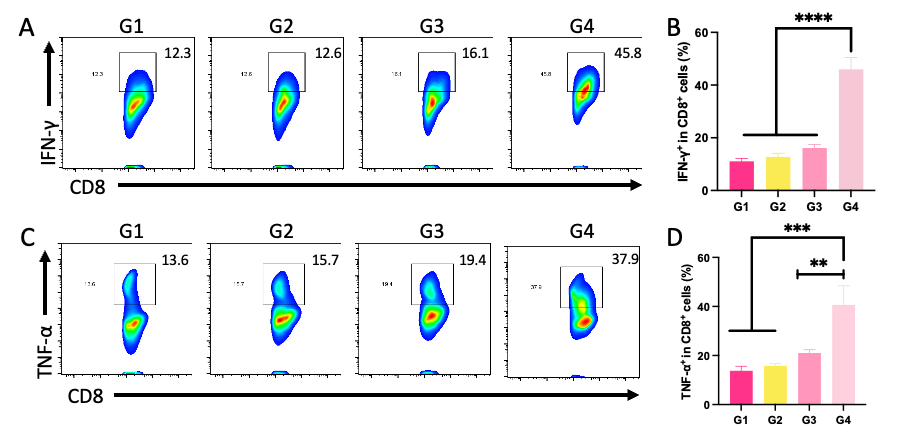
**

**Figure S16.** Representative scatter plots and histograms of the proportion of CD8+IFN-γ (A, B), CD8+TNFα+ (C, D) in the tumor on Day 17 for different treatment groups (n=3). G1: Saline, G2: Physical mixture of LP_11_, P_3_-OVA_8_, CpG and LLP2A-P_4_, G3: LP_11_[*OVA_8_/CpG*], G4: LP_11_[*OVA_8_/CpG/LLP2A*]. ** *P*<0.01, *** *P*<0.001, **** *P*<0.0001, one-way ANOVA with Turkey’s posthoc test. The mice were *s.c.* injected with different treatments, each containing an equivalent OVA_8_ concentration of 5 μM, on Days 4, 6, 9, and 12 after tumor inoculation. Tumors were collected when mice were sacrificed on Day 17.

**
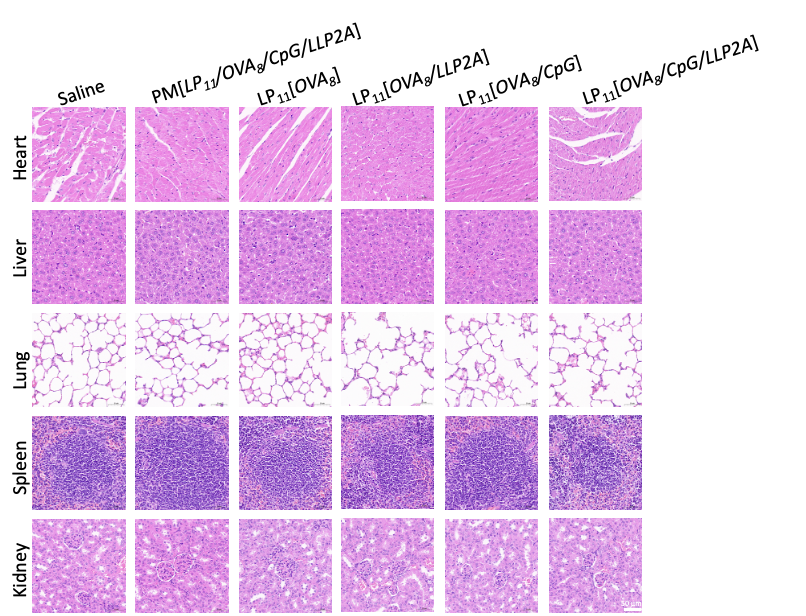
**

**Figure S17.** H&E staining of major organs in B16-OVA bearing mice after various treatments. The mice were *s.c.* injected with different treatments, each containing an equivalent OVA_8_ concentration of 5 μM, on Days 4, 6, 9, and 12 after tumor inoculation, scale bar=50 μm. The organs were collected and sectioned when mice were sacrificed on Day 17.

**Indication of the synthesized monomers by MALDI-TOF**

Figure M1 MALDI-TOF Mass Spectrometry for LP_10_: *CCCCGTAGCT.*


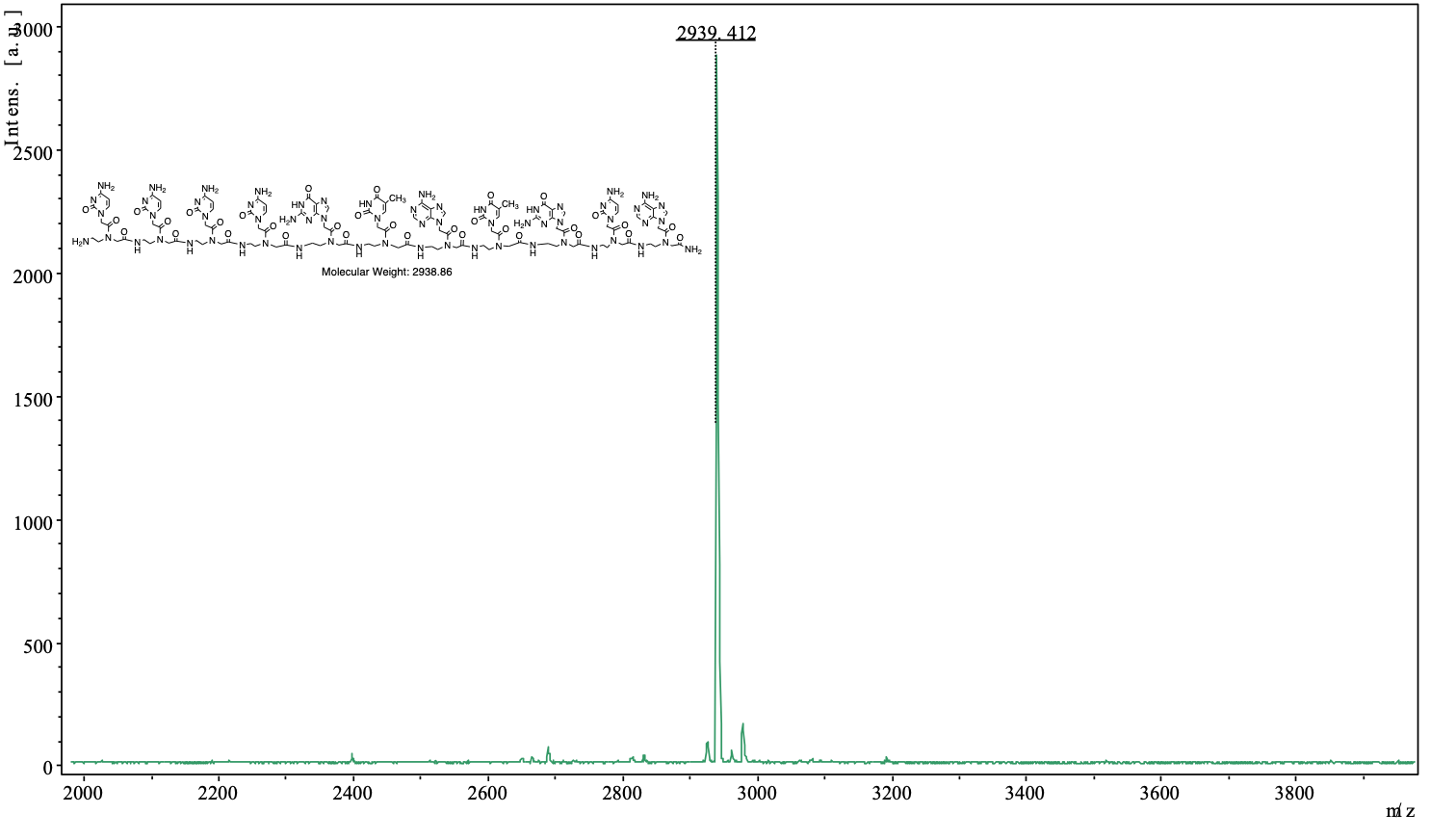


Figure M2 MALDI-TOF Mass Spectrometry for LP_11_: *CCCCGTATGCA* (M+H^+^)


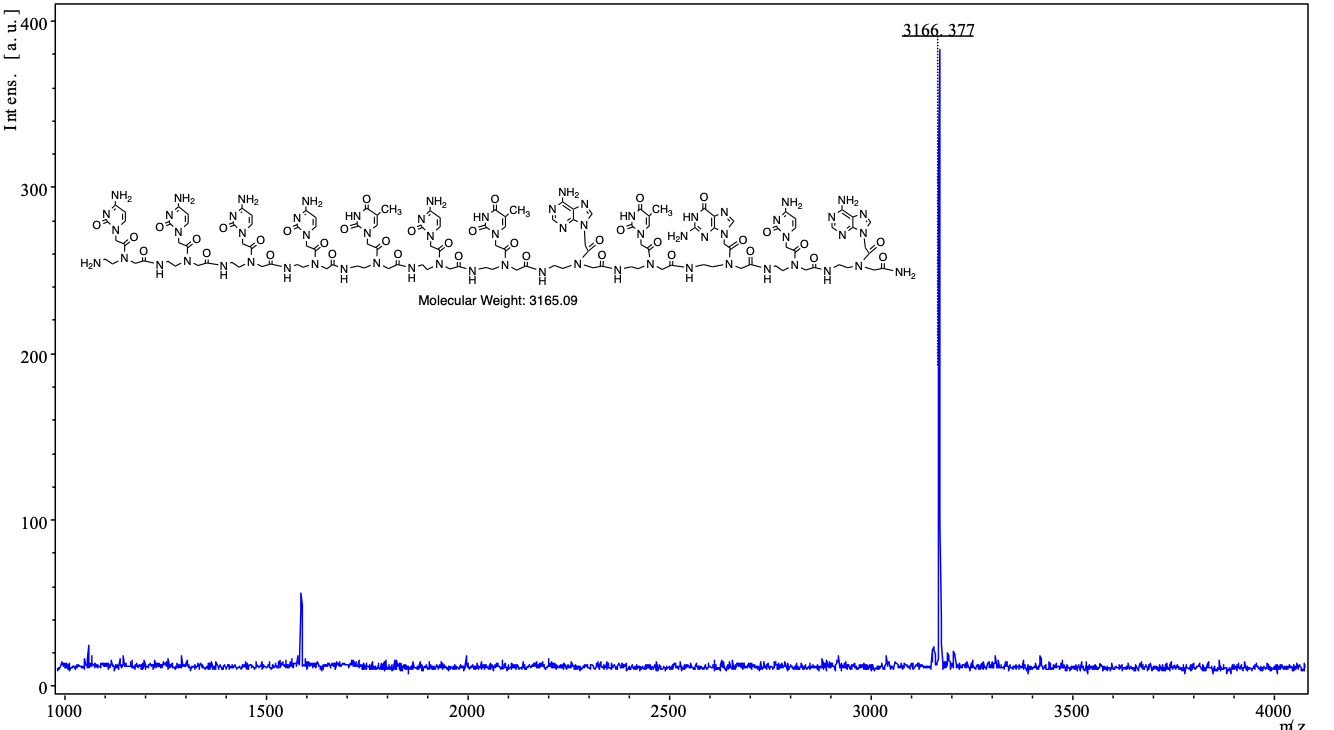


Figure M3 MALDI-TOF Mass Spectrometry for LP_12_: *CCCCTCTATGCA* (M+H^+^)


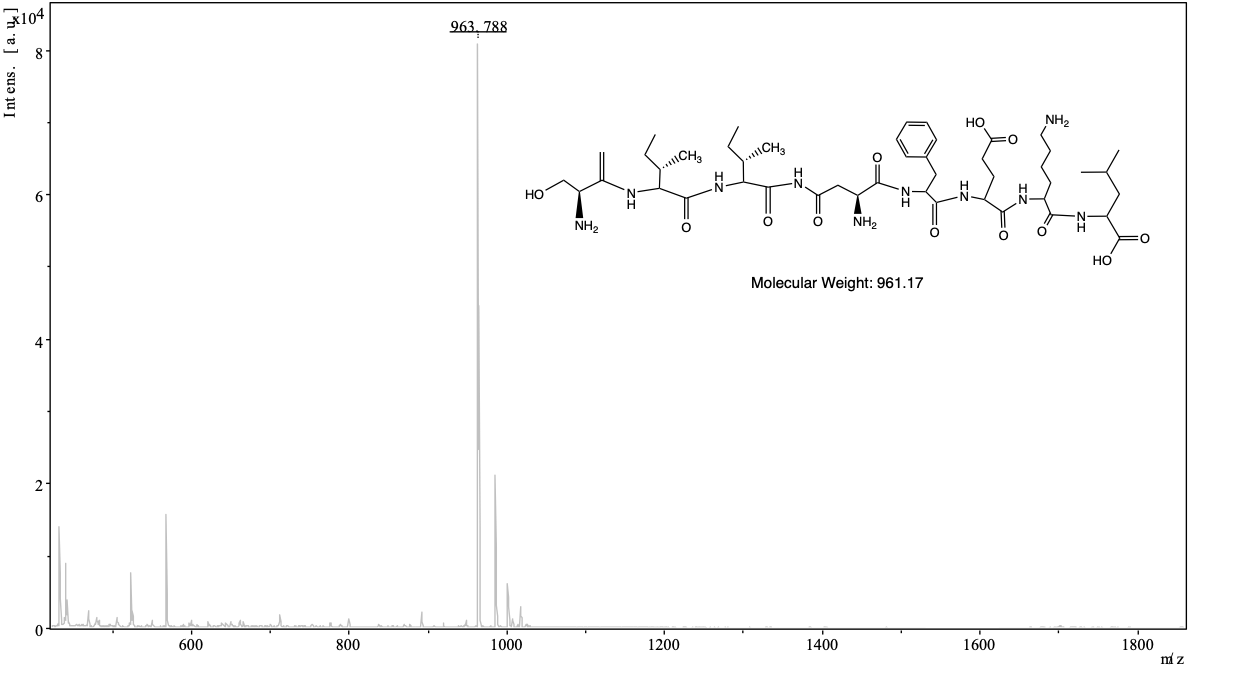


Figure M4 MALDI-TOF Mass Spectrometry for OVA_8_: SIINFEKL (M+2H^+^, M+23, M+39)


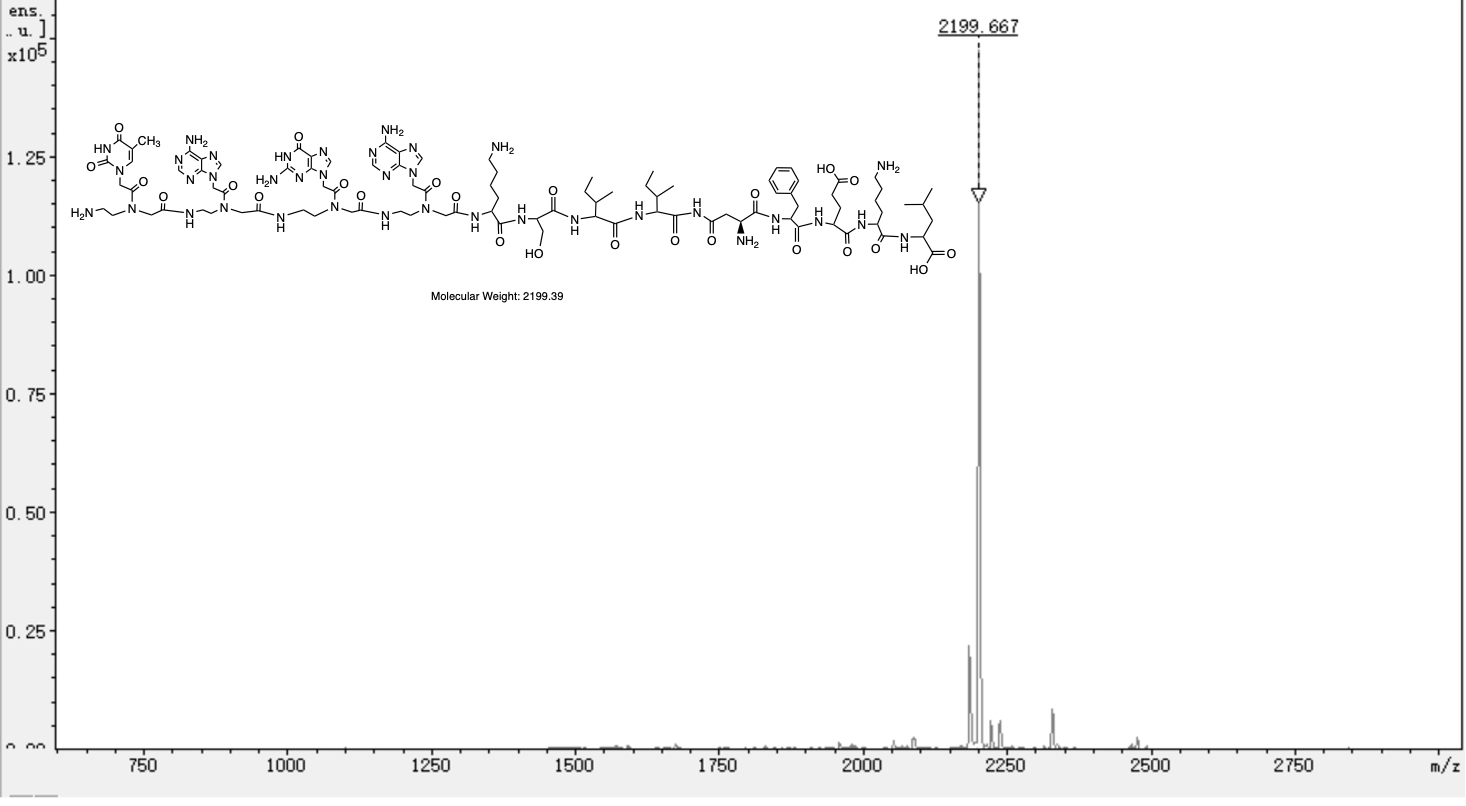


Figure M5 MALDI-TOF Mass Spectrometry for P_4_’OVA_8_: *TAGA*-K-SIINFEKL


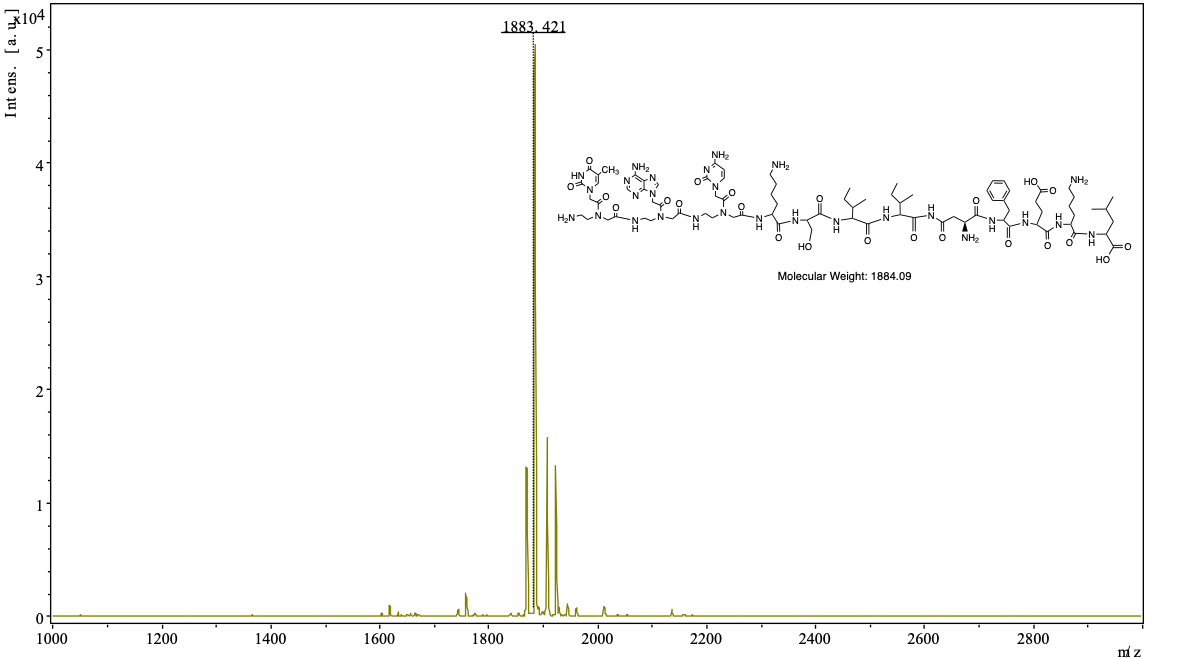


Figure M6 MALDI-TOF Mass Spectrometry for P_3_’OVA_8_: *TAC*-K-SIINFEKL (M+23, M+39)


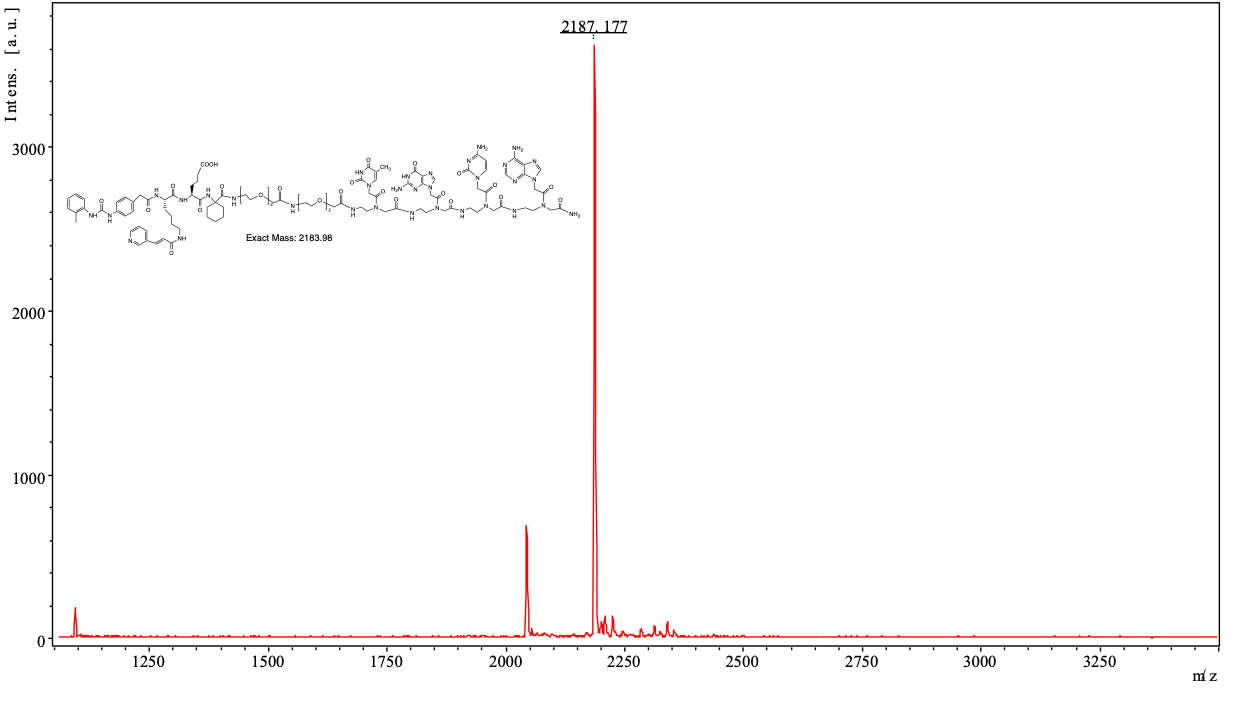


Figure M7 MALDI-TOF Mass Spectrometry LLP2A-P_4_: LLP2A-(aeea)_2_-*TGCA.*


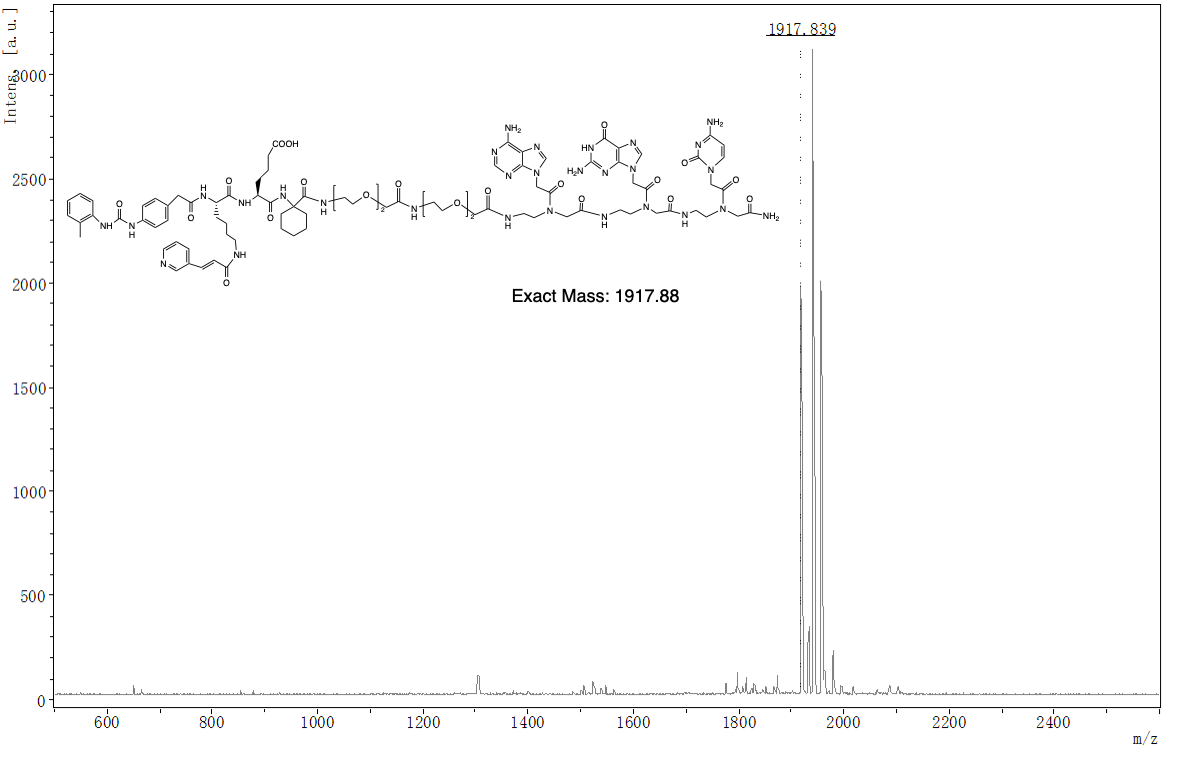


Figure M8 MALDI-TOF Mass Spectrometry LLP2A-P_3_: LLP2A-(aeea)_2_-*AGC* (M+23, M+39).


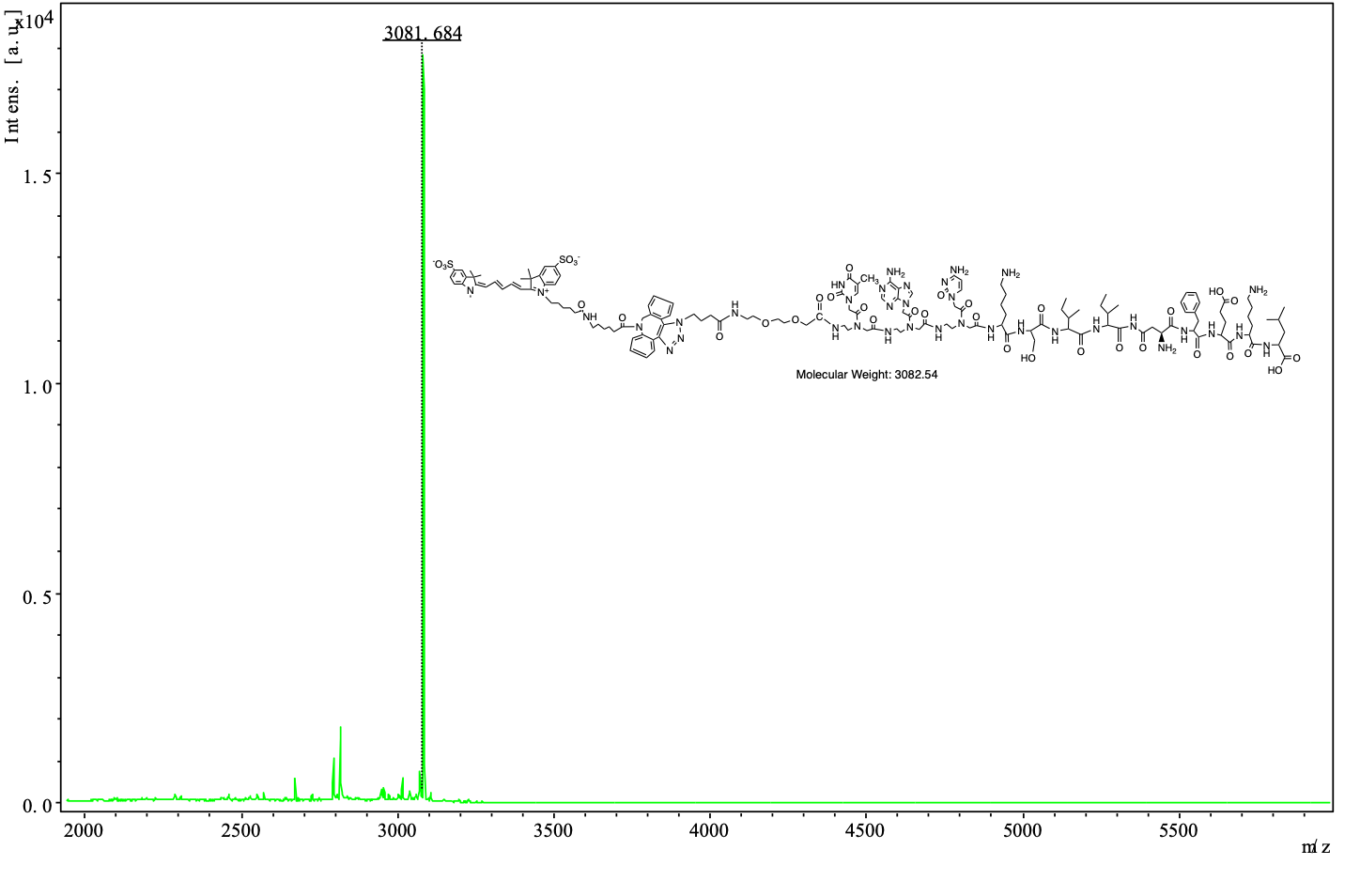


Figure M9 MALDI-TOF Mass Spectrometry for Cy5-P_3_’OVA_8_: Cy5-aeea-*TAC*-K-SIINFEKL.


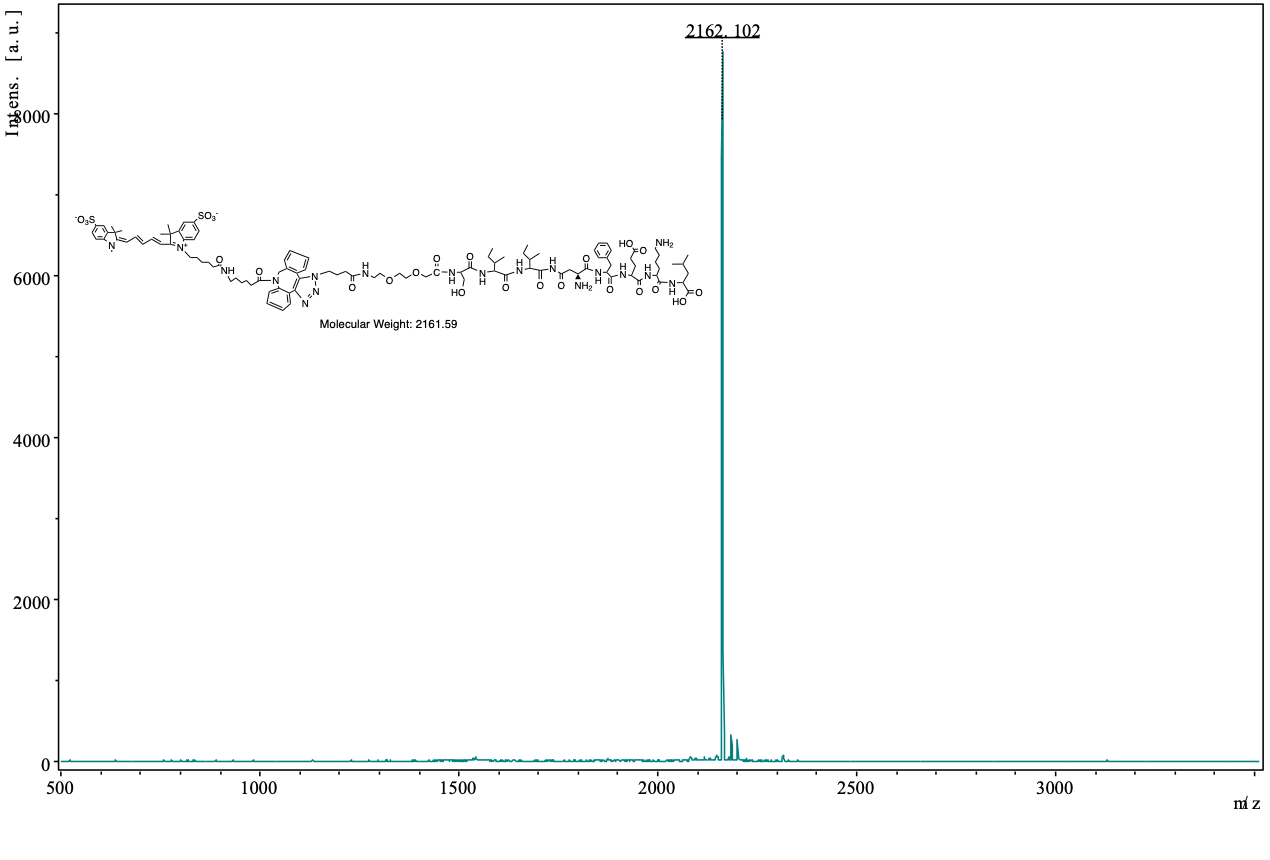
 Figure M10 MALDI-TOF Mass Spectrometry for Cy5-OVA_8_: Cy5-aeea*-*SIINFEKL.


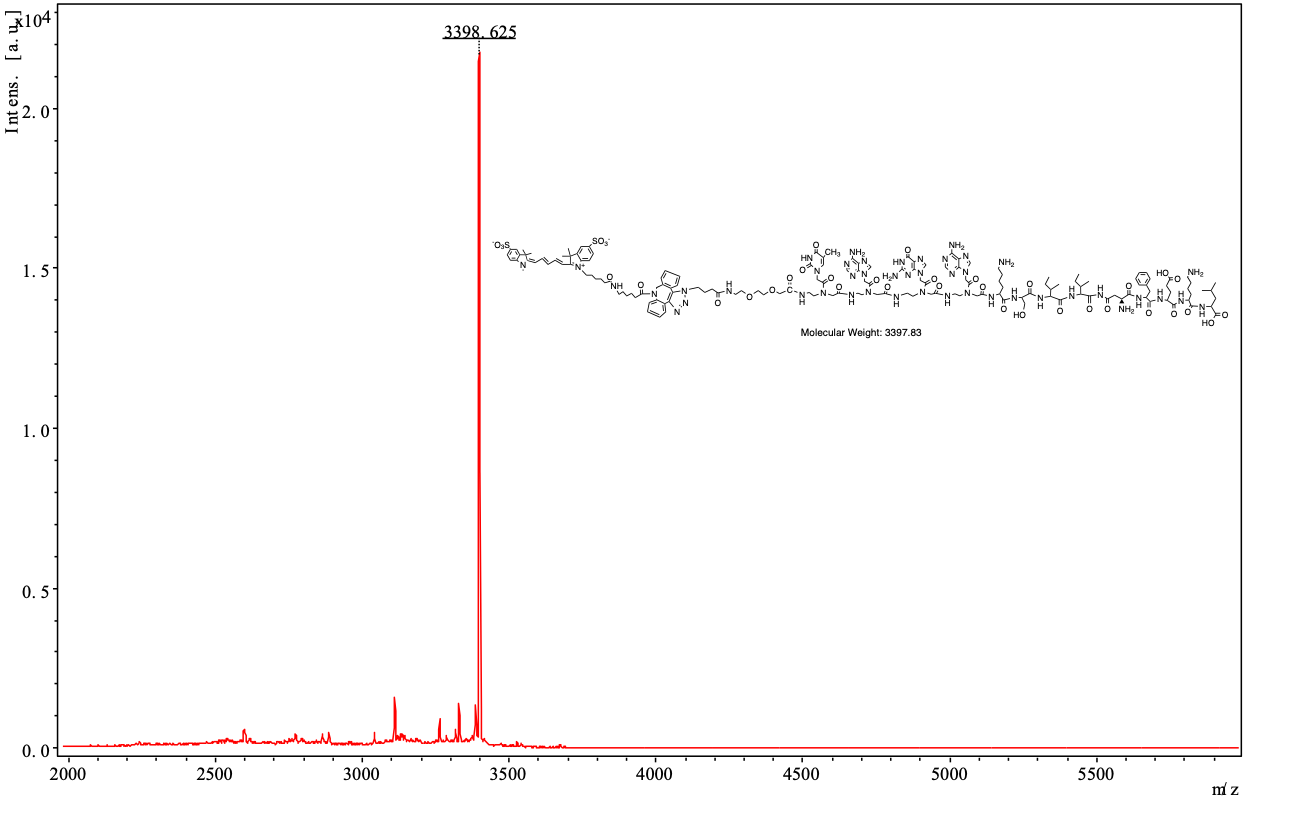


Figure M11 MALDI-TOF Mass Spectrometry for Cy5-P_4_’OVA_8_: Cy5-*TAGA*-K-SIINFEKL (M+H^+^).


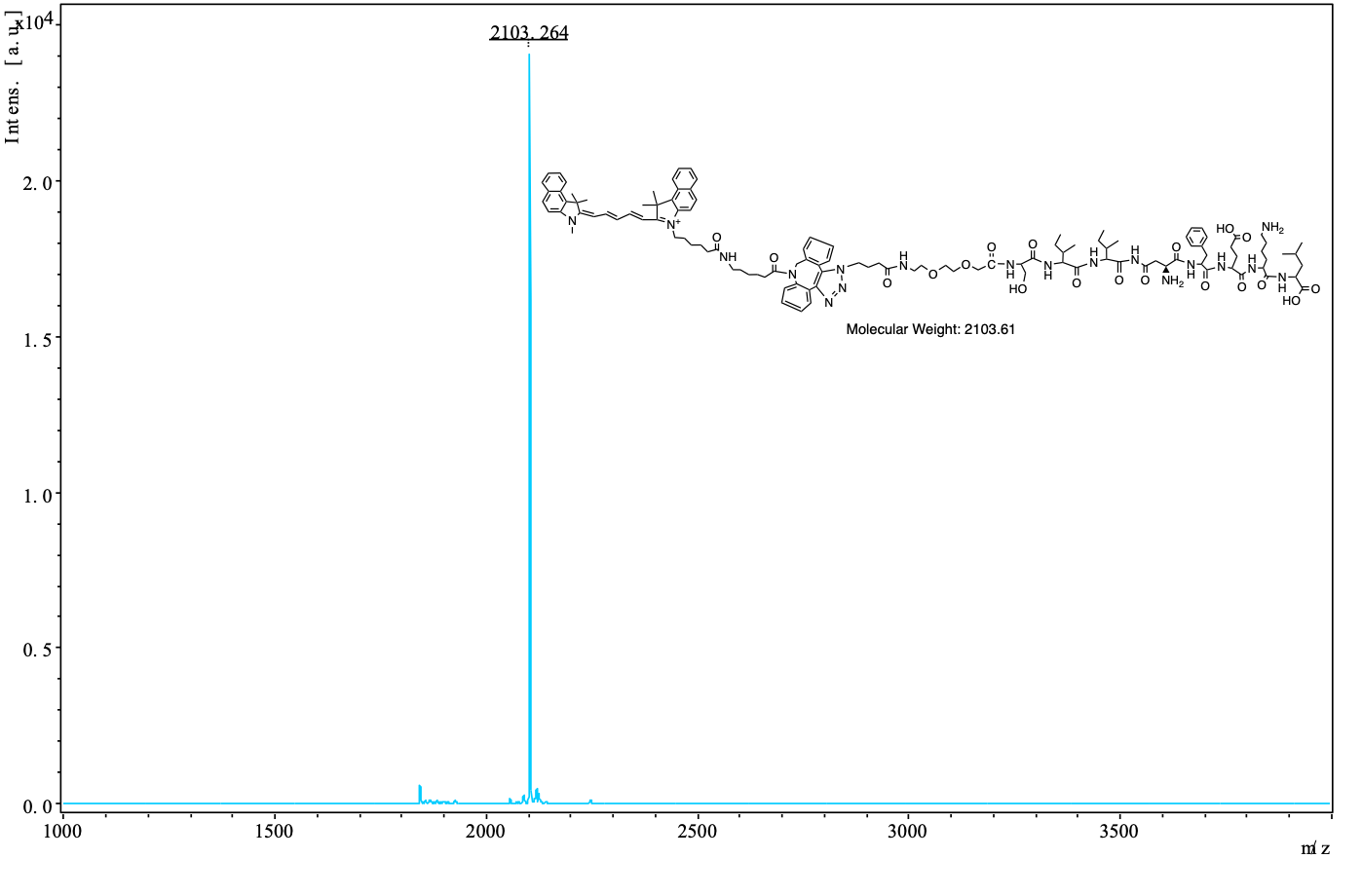


Figure M12 MALDI-TOF Mass Spectrometry Cy5.5-aeea-SIINFEKL.


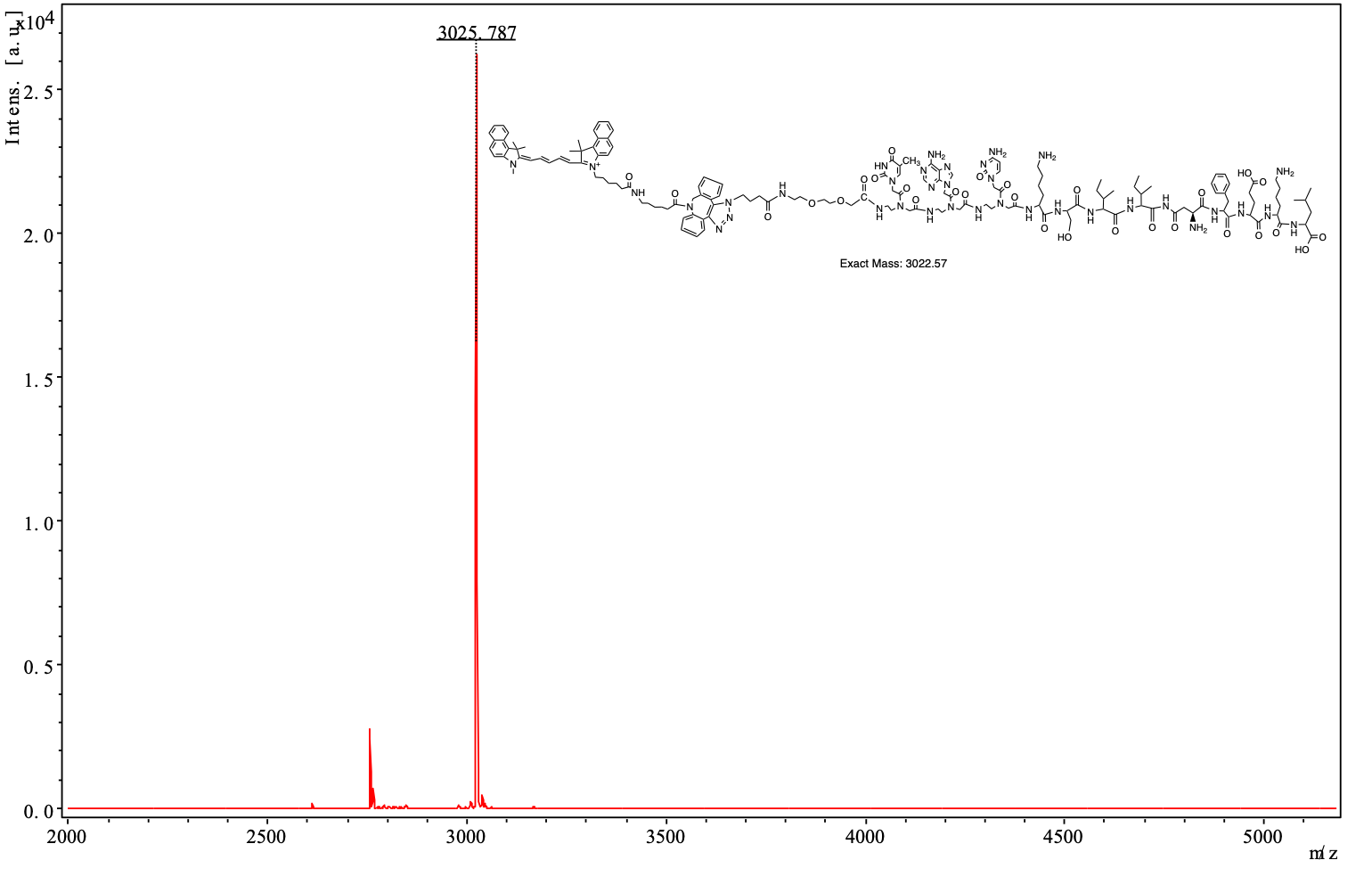


Figure M13 MALDI-TOF Mass Spectrometry for Cy5.5-P_3_’OVA_8_: Cy5.5-aeea-*TAC*-K-SIINFEKL (M+3H^+^).

**References：**

1. Wang, J.; Dai, X.; Hsu, C.; Ming, C.; He, Y.; Zhang, J.; Wei, L.; Zhou, P.; Wang, C. Y.; Yang, J.; Gong, N. Discrimination of the heterogeneity of bone marrow‑derived dendritic cells. *Mol Med Rep.* 2017; **16** (5), 6787-6793.

2. Tan, Y. S.; Lei, Y. L. Isolation of Tumor-Infiltrating Lymphocytes by Ficoll-Paque Density Gradient Centrifugation. *Methods Mol Biol.* 2019; **1960**, 93-99.

3. Wang, D.; Nie, T. Q.; Huang, C. Q.; Chen, Z. R.; Ma, X. C.; Fang, W. M.; Huang, Y. Y.; Luo, L. P.; Xiao, Z. Y. Metal-Cyclic Dinucleotide Nanomodulator-Stimulated STING Signaling for Strengthened Radioimmunotherapy of Large Tumor. *Small.* 2022; **18** (41), e2203227.
